# Seeing through experts' eyes: a foundational vision-language model trained on radiologists' gaze and reasoning

**DOI:** 10.1038/s44387-026-00136-9

**Published:** 2026-07-13

**Authors:** Kinhei Lee, Peiyuan Jing, Zhenxuan Zhang, Yue Yang, Tao Wang, Dominic C. Marshall, Yingying Fang, Guang Yang

**Affiliations:** 1 Bioengineering Department and Imperial-X, Imperial College London, London, UK; 2 College of physics and information engineering, Fuzhou University, Fuzhou, China; 3 Department of Surgery and Cancer, Imperial College London, London, UK; 4 National Heart and Lung Institute, Imperial College London, London, UK; 5 Cardiovascular Research Centre, Royal Brompton Hospital, London, UK; 6 School of Biomedical Engineering & Imaging Sciences, King’s College London, London, UK

**Keywords:** Computational biology and bioinformatics, Health care, Mathematics and computing, Medical research

## Abstract

Large-scale vision-language models have shown promise in automating chest X-ray interpretation. However, their clinical utility remains limited, since most systems optimize for semantic information rather than emulating how experts visually examine and interpret medical images. As a result, current models often overlook critical findings, misrepresent anatomical context, or diverge from established diagnostic workflows. Radiologists, by contrast, follow structured protocols that sequentially assess anatomical regions, reducing missed findings and supporting reliable diagnostic reasoning. We therefore introduce Gaze-X, a vision-language model that leverages radiologists’ eye-tracking data as a behavioral prior for expert diagnostic reasoning. By incorporating gaze trajectories and fixation patterns into pretraining, Gaze-X learns to follow the spatial and temporal structure of radiologist attention. Using a curated dataset of over 30,000 key frames from five radiologists interpreting chest X-rays across diverse disease categories, we show that Gaze-X produces more accurate, interpretable, and expert-consistent outputs across a range of clinically relevant tasks. Unlike autonomous reporting systems, Gaze-X produces verifiable evidence artifacts, including inspection trajectories and finding-linked localized regions, enabling transparent and safe human-AI collaboration. This capability provides a practical route toward more trustworthy, explainable, and diagnostically robust AI for radiology and beyond.

## Introduction

With the exponential growth in medical imaging volume and recent advances in multimodal generative artificial intelligence (AI), automated report generation from chest X-rays has become an increasingly important direction in clinical AI^1–3^. Such systems have the potential to reduce radiologists’ workload and improve the efficiency of medical reporting^1,2,4,5^. While recent models demonstrate promising fluency in free-text generation, radiology reports are not merely descriptive narratives. They are structured diagnostic documents that reflect a step-by-step reasoning process grounded in anatomical and pathological interpretation. To ensure clinical reliability and interpretability, generated reports should follow established conventions in how radiologists observe, organize, and describe findings^6,7^. This means that clinically useful and trustworthy report generation goes beyond image-text alignment. It must also follow standardized diagnostic protocols of clinicians^8^.

**Fig. 1:**
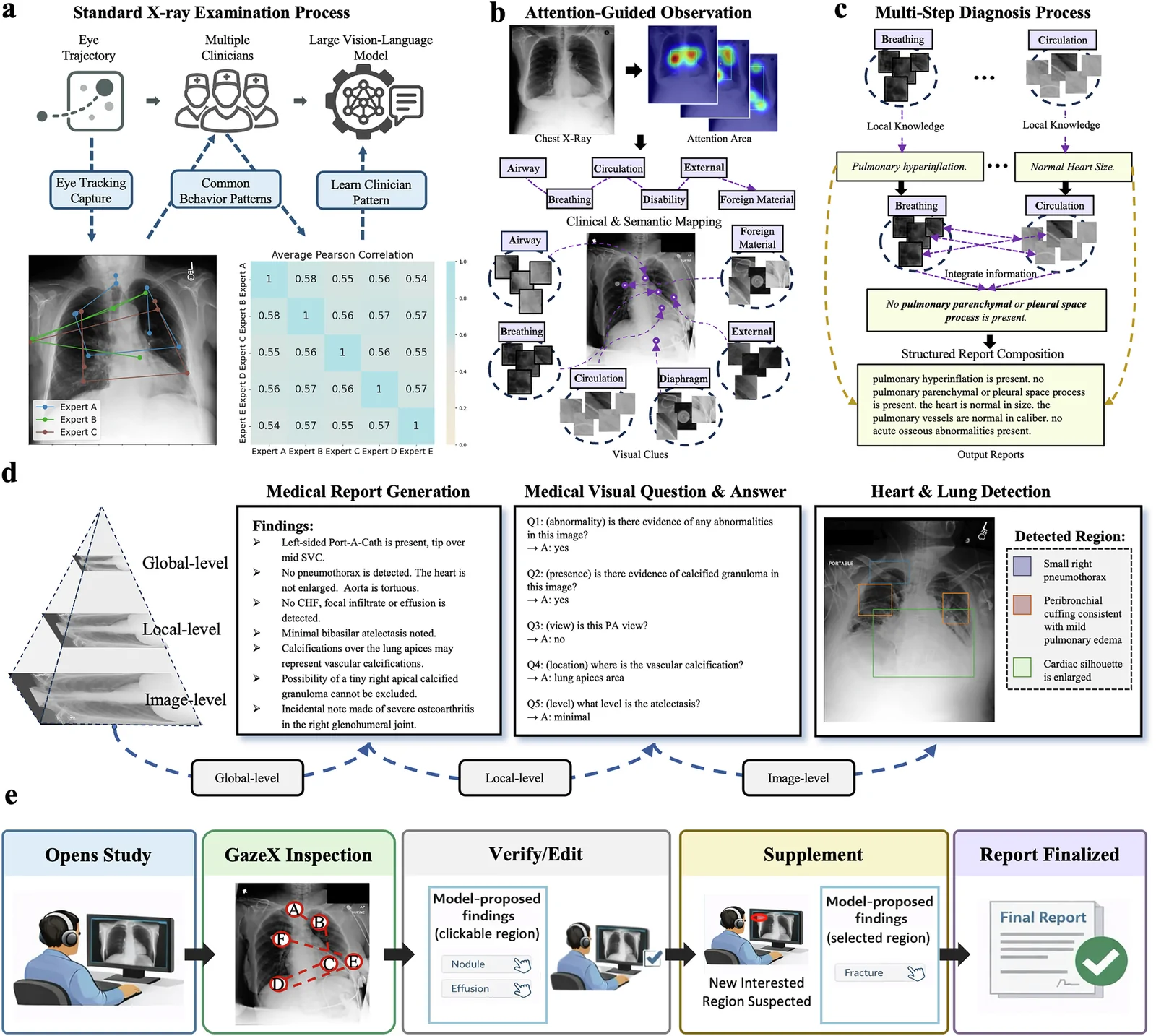
Radiologist-guided diagnostic reasoning patterns for structured chest X-ray interpretation. **a** Radiologists' gaze behaviors demonstrate structured and reproducible diagnostic workflows. Eye-tracking captures fixation trajectories from multiple experts, which reveal common attention patterns aligned with established reading protocols of clinicians. Statistical analysis of inter-expert trajectories shows consistent correlations, highlighting shared visual strategies during chest X-ray interpretation. Enabling large vision-language models to learn such standardized reading workflows has the potential to enhance the performance across diverse clinical downstream tasks. **b** Attention-guided visual observations align with clinically defined categories (i.e., airways, breathing, circulation, diaphragm, external devices, and foreign materials), thereby providing a semantic mapping between image features (visual clues from different regions within the examined area) and diagnostic reasoning. **c** Visual clues from key anatomical regions are integrated during multi-step diagnostic processes to support structured report generation. **d** Downstream tasks such as segmentation, visual question answering (VQA), and report-generation benefit from the model’s alignment with expert gaze. This figure illustrates how expert eye-movement data capture the temporal and spatial logic of radiological decision-making, serving as supervision signals to guide AI models toward clinically grounded and interpretable report generation. **e** Clinician-in-the-loop workflow enabled by GazeX. GazeX provides inspection cues and finding-linked regions to support systematic review and human verification, allowing radiologists to verify or edit model suggestions before finalizing the report.

As shown in Fig. 1, standardized guidelines for interpreting medical images are essential in clinical practice, as they ensure consistency across clinicians, support effective communication, and reduce the risk of overlooking critical findings^9,10^. In real practice, radiologists employ a systematic approach when interpreting chest X-ray images, often following the classic ABCDEF guideline (i.e., Airways, Breathing, Circulation, Diaphragm, External and Foreign material) to (1) minimize missed findings, (2) ensure diagnostic consistency, and (3) identify the patient’s most life-threatening problems first^11,12^. Within this framework, specific observation patterns are followed for each area. For example, the lungs are divided into three zones (upper, middle, and lower), where each occupies about one-third of the lung’s height. Each zone is inspected from top to bottom for lung markings, which appear as small, faint white lines. Inspecting each zone in sequence helps radiologists systematically check for irregularities, such as missing markings, which may indicate a pneumothorax. More importantly, the sequential viewing approach, progressing from the entire lung to smaller subsections, allows radiologists to first assess whether the lung is uniformly expanded. When abnormalities are detected, radiologists can examine smaller anatomical regions to determine the underlying cause and confirm how extensive the pathology is. These examinations provide essential visual clues to verify and consolidate the diagnosis^11^. These viewing patterns reflect how radiologists allocate visual attention during image interpretation^13^. Recent studies have demonstrated that eye gaze trajectories are not random but closely follow clinical reasoning frameworks^8,14^. However, current AI models lack this attention alignment. Incorporating a radiologist's eye gaze into training may help AI systems better emulate human diagnostic workflows and generate more clinically aligned reports.

Although many automated chest X-ray interpretation systems have emerged in recent years, they still face critical challenges in achieving the level of clinical reliability and consistency expected in real-world settings^5,8,15–18^. These challenges largely stem from the mismatch between how radiologists interpret medical images and how current AI models are designed^15,19,20^. One major limit is the lack of clear alignment between the model’s diagnostic outputs and the corresponding anatomical regions in the image (e.g., when asked to identify pneumothorax, a system might return a positive response without indicating any visual evidence). The system’s conclusions remain opaque and difficult to verify without grounding its answers in observable image features^21–23^. Another concern is whether the system thoroughly examines all relevant areas that a radiologist would routinely inspect. Radiologists are trained to follow a structured workflow, which includes checking the lung zones, heart borders, diaphragm contours, costophrenic angles, and soft tissues. Many AI systems do not have mechanisms to ensure such comprehensive coverage, often omitting regions like the lower lung fields where early signs of pathology (e.g., pleural effusions or basal infiltrates). This kind of partial examination can easily result in missed or inaccurate findings. In addition, current systems often lack the ability to integrate information across multiple regions of the image. Radiological diagnosis commonly requires synthesizing information from several anatomical areas^24^. For instance, differentiating pulmonary edema from pneumonia often involves evaluating the symmetry of lung opacities, measuring the size of the cardiac silhouette, and assessing vascular congestion patterns. Without modeling such contextual dependencies, automated systems struggle to make accurate and coherent diagnostic judgments. These issues collectively raise concerns about the clinical applicability of end-to-end AI systems that operate without constraints. Therefore, it is essential to incorporate the standardized examination patterns and diagnostic reasoning processes that radiologists follow in practice into the design and training of automated systems to ensure safe and trustworthy deployment.

In this paper, we propose the GazeX framework. It leverages radiologists’ eye-tracking data to model the standard spatial and temporal dynamics of expert image interpretation. This helps bridge the gap between current AI models and expert radiologist behavior. Here, we define expert behavior as the patterns that are common across radiologists’ examinations (i.e., the shared gaze trajectories associated with a given disease and its corresponding anatomical region). We interpret these shared patterns as a manifestation of the common expert knowledge that radiologists implicitly rely on during diagnostic reading. The core idea of GazeX is to treat these eye-movement patterns as primary supervision signals that reflect clinically grounded reasoning strategies. During pretraining, the model is guided to attend to image regions in a way that mirrors both components of radiologists’ gaze: the spatial attention (which regions they focus on) and the temporal sequence (the order in which their focus shifts between regions). This enables the system to learn not only which areas are diagnostically important, but also how attention evolves over time, as a reasoning process, and is integrated into a final diagnostic decision. Compared to prior methods that focus on aligning generated text with report content or rely on region-level attention maps derived from class activation, GazeX is explicitly trained to reproduce expert gaze behavior. Our model internalizes detailed visual attention trajectories collected during real diagnostic workflows. This allows GazeX to move beyond the surface-level and rough alignment of free-form radiology reports with chest X-ray images and to approximate the deeper visual reasoning employed by radiologists. Moreover, while some prior studies have explored eye-tracking for prior information, our approach is one of the first attempts to incorporate it directly into the model’s learning objective during pretraining. By embedding expert behavioral signals into the foundation of model training, GazeX offers a new paradigm for designing clinically grounded diagnostic systems. Rather than treating expert knowledge as a static label or external constraint, our method learns from how experts observe, integrate visual evidence, and reason through images during routine diagnosis. By aligning model behavior with established radiological workflows, this approach enables AI outputs to be reviewed and verified in relation to familiar reading steps and anatomical regions. This further facilitates clinician oversight and helps identify potential omissions. As a result, GazeX supports more transparent and interpretable human-AI interaction, providing a practical pathway toward trustworthy and expert-aligned AI systems for diagnostic radiology. As shown in Fig. 1, we aim to model radiologists’ systematic visual inspection behavior to support clinician-in-the-loop chest X-ray interpretation. (1) We analyzed radiologists’ eye-tracking data to identify common patterns in how experts systematically inspect a chest X-ray image during diagnosis. This study involved five radiologists viewing the same chest X-ray images across 41 cases, with their fixations and trajectory correlations measured, revealing consistent inspection strategies across clinically relevant regions. (2) We leveraged curated eye-tracking data comprising over 30,000 key frames spanning diverse disease categories to explicitly supervise model training in both temporal and spatial dimensions. This supervision enables the model to follow sequential attention patterns and to associate each reported finding with localized visual evidence, producing verifiable inspection trajectories and region-level evidence that can be audited by clinicians. (3) We evaluated whether the model reads and interprets chest X-ray images in a manner consistent with expert radiologists, and whether enforcing systematic, inspection-trajectory constraints during model training improves coverage and reliability in downstream applications, as opposed to reliance on ungrounded shortcuts. These applications comprise a total of 231,835 radiographic studies with associated reports, 780,014 question-answer pairs, and 1162 image-sentence pairs containing bounding boxes and corresponding phrases, spanning varying levels of complexity.

## Results

### GazeX performance in radiology report generation

**Fig. 2:**
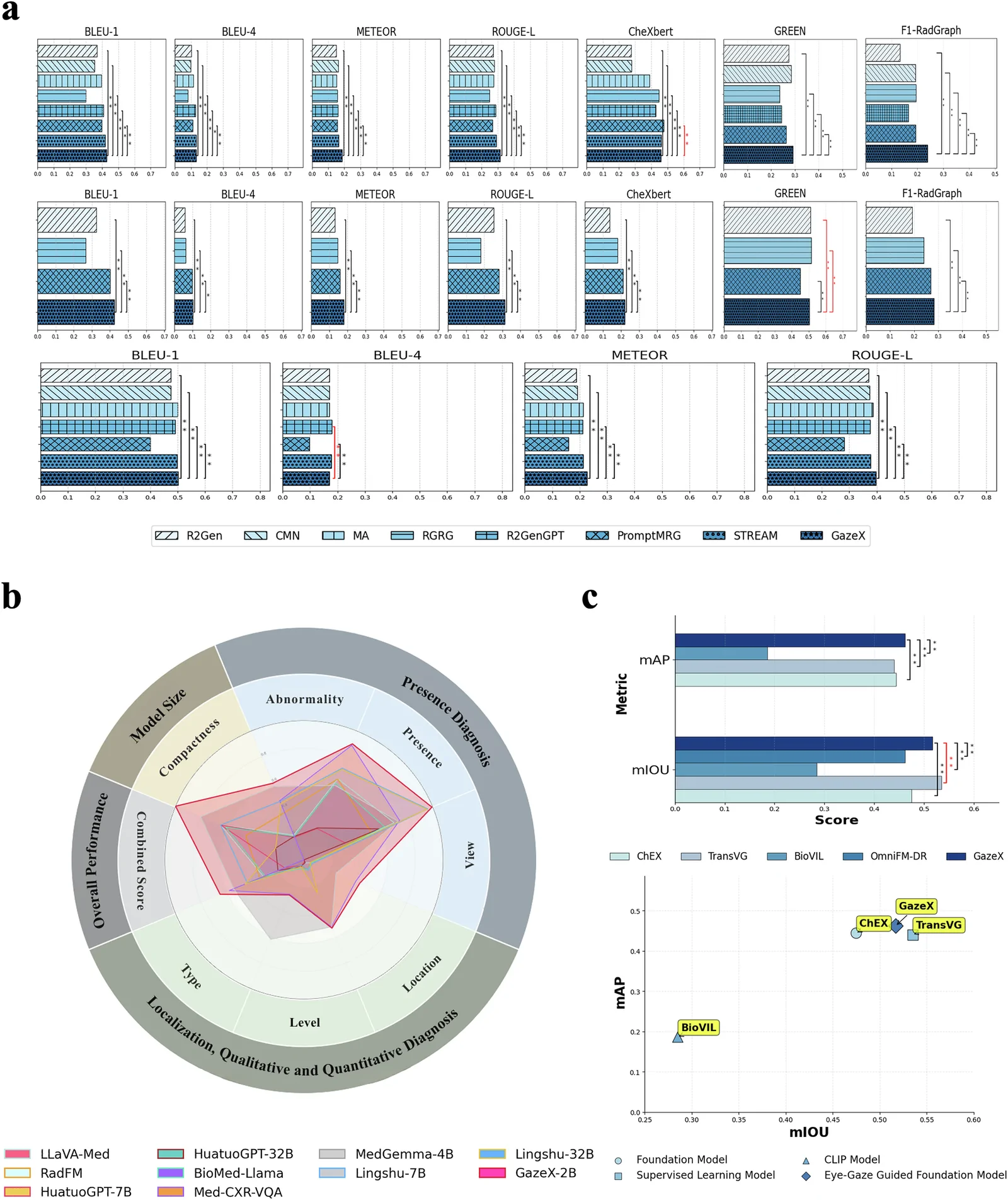
Evaluating GazeX performance on chest radiology report generation, visual question answering and visual grounding. **a** Comparison of different report-generation methods across various evaluation metrics in MIMIC-CXR^42^ and IU X-ray^43^. The shown results include evaluations on the MIMIC-CXR testing set using the GazeX model fine-tuned on MIMIC-CXR, zero-shot evaluations on the entire IU X-ray dataset using the GazeX model fine-tuned on MIMIC-CXR, and evaluations on the IU X-ray testing set using the model fine-tuned on the IU X-ray dataset. The Wilcoxon signed-rank test was performed to assess the statistical significance of the improvements compared to other models. ** indicates *p* < 0.01, * indicates *p* < 0.05, and ** indicates the reverse situation. **b** Radar chart showing foundation models' performance on different perspectives of the visual question answering dataset, including model size, overall performance, localization, qualitative and quantitative diagnosis aspects, and presence diagnosis aspects. **c** Quantitative analysis, including mean Intersection over Union (mIOU) and mean Average Precision (mAP), of the accuracy of the visual grounding task across all disease categories for different model types (e.g., foundation models, CLIP-based models and generative models). Additional quantitative comparisons are provided in Supplementary Tables 1–3.

GazeX learns to emulate radiologists’ global examination patterns, allowing it to integrate holistic visual information from the X-ray image for diagnosis and effectively transform these observations into free-form radiology reports. We conducted evaluation of GazeX report-generation ability on two datasets: MIMIC-CXR (77,110 images and 227,835 reports) and IU X-ray (7470 images and 3,955 reports). For the IU X-ray dataset, we evaluated two model versions: (1) GazeX model fine-tuned on the IU X-ray dataset and (2) zero-shot inference using the GazeX model pretrained on MIMIC-CXR. The findings from^25^ indicated that the official test split of the IU X-ray dataset is unsuitable for disease-specific evaluation due to insufficient positive samples for certain diseases. Given these limitations, for IU-Xray, clinical accuracy measured by CheXbert was assessed using the complete dataset instead of the designated test split for model version 2. As shown in Fig. 2a, GazeX demonstrates outstanding performance in NLG metrics, achieving state-of-the-art performance in BLEU and ROUGE-L scores across two report-generation datasets: MIMIC-CXR (0.427 and 0.315) and the IU X-ray setting (1) (0.502 and 0.398). The model performance is stable across two public datasets with consistent improvements in BLEU-1 (0.427 in MIMIC-CXR and 0.502 in the IU X-ray setting (1)) and BLEU-2 (0.277 in MIMIC-CXR and 0.333 in the IU X-ray setting (1)) while maintaining BLEU-3 and BLEU-4 performance comparable to the state-of-the-art models, such as STREAM^26^. Using the CheXbert-based F1 score as a factual accuracy metric, GazeX shows promising performance in generating clinically accurate radiology reports, achieving CheXbert F1 scores of 0.460 on MIMIC-CXR and the highest reported performance on IU X-ray (setting 2), highlighting its robust performance on the external dataset not used in training. To further assess clinical accuracy beyond lexical overlap, we additionally report GREEN^27^, an LLM-based evaluation framework, and RadGraph-F1^28^, which computes the F1 agreement between clinical entity-relation graphs extracted from the generated and reference reports. As shown in Fig. 2a, GazeX achieves statistically significant improvements on both datasets, MIMIC-CXR (0.256) and IU X-ray setting (2) (0.282), in terms of the RadGraph-F1 metric, as well as on the MIMIC-CXR (0.293) setting under the GREEN score.

### GazeX performance in visual-question answering

GazeX not only learns to replicate the radiologist’s global view analysis for a holistic diagnosis, but also adapts to targeted tasks by examining specific image regions for answering visual questions that require localized observations. The evaluation of visual-question answering is divided into six categories, Abnormality, Presence, Location, Level, Type, and View, each evaluating different levels of detail^29^. For example, in chest radiograph analysis, multiple questions are posed when an abnormality is detected, including its type, severity, and anatomical localization. In the case of pulmonary edema, three questions arise: (1) the presence of edema, (2) its anatomical distribution, and (3) the degree of severity. Addressing these questions requires localized feature extraction and detailed observational analysis. As shown in Fig. 2b, GazeX demonstrates significant performance compared to established foundation models. We selected models pretrained on large-scale medical imaging datasets, including both exclusively radiological images and those with broader medical imaging data. While state-of-the-art foundation models like MedGemini, LLaVA-Med, and RadFM are pretrained on large-scale medical image-text datasets covering diverse tasks, GazeX utilizes radiologists’ inspection patterns to achieve significant performance gains. When targeting specific diseases, GazeX can identify the anatomical regions that radiologists are most likely to examine. Furthermore, the model captures the comparative examination strategy employed by radiologists, who typically observe both abnormal areas and corresponding normal regions to establish diagnostic evidence and assess disease severity and presence. By learning from these visual inspection patterns, GazeX can more precisely identify abnormalities, severity levels and anatomical locations. GazeX exhibits significant improvements across multiple diagnostic categories quantitatively, including abnormality detection, presence identification, view classification, and anatomical localization. The model achieves the highest combined performance with a top-1 accuracy of 0.651, demonstrating the effectiveness of incorporating expert visual attention patterns in medical visual question answering tasks. In particular, when answering abnormality-related questions that require visual feature extraction from multiple regions and comparative analysis of normal and abnormal areas within chest radiographs, GazeX demonstrates state-of-the-art performance. Pretrained in radiologist examination patterns, the model achieves a top-1 accuracy of 0.594 in abnormality identification and 0.902 in abnormality presence detection by effectively integrating both global and local features for accurate diagnostic reasoning. Global features encompass comparative analysis between the fields of the left and right lung to detect abnormalities such as edema, while local features focus on the detailed examination of specific abnormal regions within affected lung areas. Importantly, presence detection represents the foremost clinical priority, as missed abnormalities can have direct consequences for patient care. Given that GazeX was designed to assist radiologists, its performance in this domain suggests that it may provide meaningful support in reducing errors at the critical first step of abnormality detection and improving the reliability of radiological assessment.

### GazeX performance in visual grounding

To perform a visual grounding task based on radiologists’ findings, different types of methods are compared, including generative models^30^, CLIP-based models^31^, foundation models^32^, and supervised bounding box learning models^33^. Our approach surpasses the foundation and CLIP-based models by an average of 42% in mIOU and 60% in mAP. During pretraining, GazeX learns to perform comparative analysis, contrasting normal and pathological regions or evaluating features across anatomical sub-structures such as lung lobes, to inform its diagnostic decisions, as further demonstrated in the Discussion (GazeX reproduces radiologist inspection patterns). Because pretraining relies on Gaussian heatmaps generated from radiologists’ fixation points (see Methods), the supervised attention region is inherently broader than a tight bounding box (see Enhancing diagnostic detail and spatial reasoning with inspection-guided modeling in the Discussion). As a result, when fine-tuned for visual grounding tasks, GazeX tends to attend to a wider region than ground-truth annotations. Despite this, our model achieves performance comparable to fully supervised methods that are explicitly trained to align radiologists’ findings with annotated bounding boxes.

### GazeX performance across downstream tasks

The improvements of GazeX are both clinically meaningful and statistically significant across downstream tasks. Beyond text-overlap measures, we also evaluate on clinically grounded metrics, where our model continues to demonstrate outstanding performance: CheXbert, which measures agreement on 14 clinically actionable findings used in radiology practice (GazeX: 0.460); RadGraph-F1 (GazeX: 0.256)^28^, the F1 agreement between clinical entity-relation graphs extracted from generated and reference reports; and GREEN (GazeX: 0.293)^27^, an LLM-based clinical evaluation framework (Fig. 2a). For visual question answering, we report top-1 accuracy on clinically relevant questions, disease presence, severity, and anatomical location, reaching a combined score of 0.651. GazeX achieves state-of-the-art performance on visual question answering, report generation, and visual grounding, and these gains are statistically significant over competing models (Wilcoxon signed-rank test, *p* < 0.05).

## Discussions

### Radiologists exhibit consistent inspection patterns

We leverage radiologist eye-tracking data collected during chest X-ray interpretation to pretrain GazeX with human-aligned visual supervision. To examine whether radiologists follow consistent inspection strategies, we analyzed 41 frontal chest X-ray images, each independently reviewed by five radiologists who provided clinical descriptions while their eye movements were tracked. Since raw gaze recordings may include fixations that fall outside the image boundaries, we further applied a filtering step that excluded these off-screen points.

**Fig. 3:**
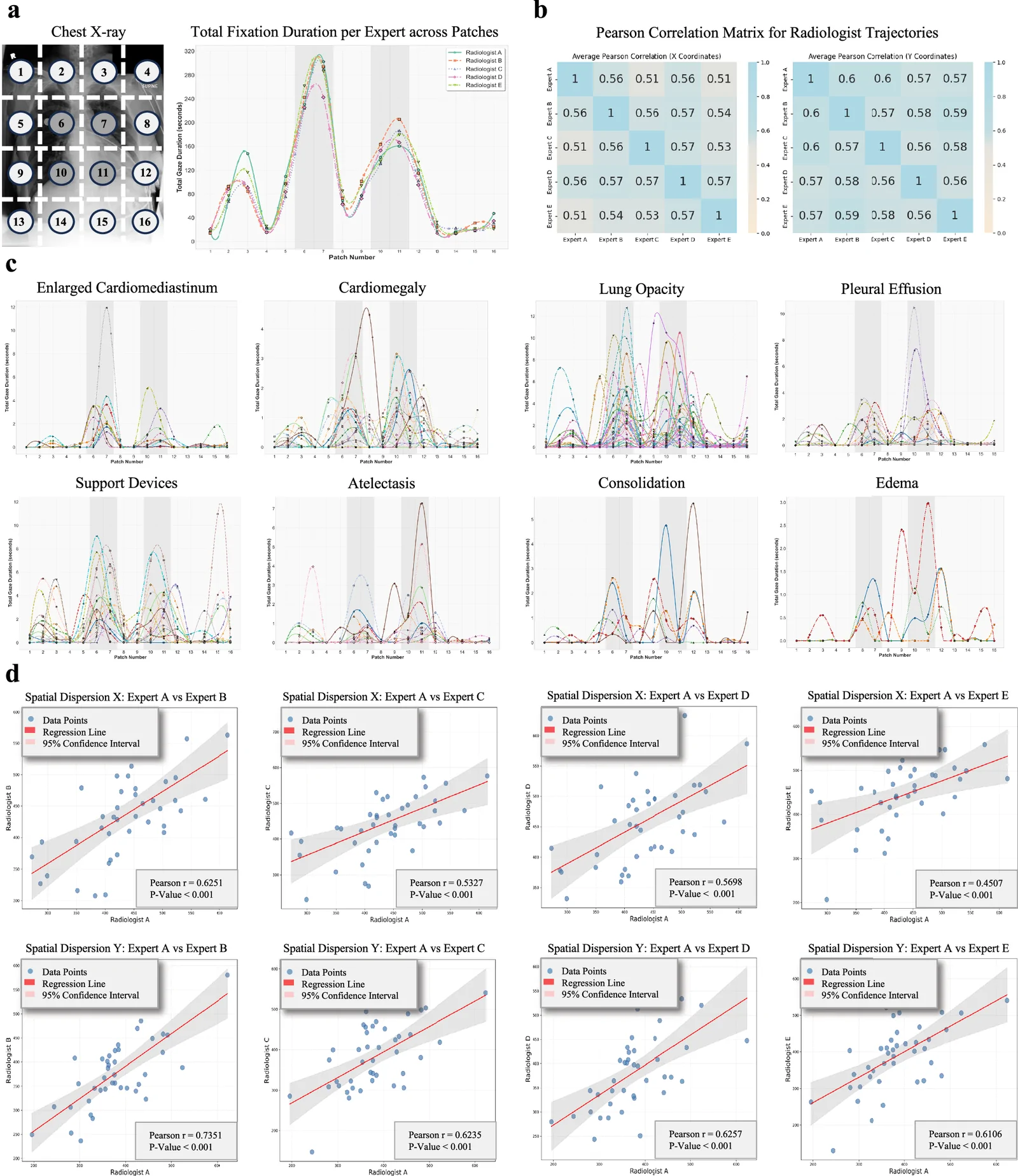
Consistent inspection patterns among radiologists. **a** Total time spent per radiologist across a 4 × 4 grid of patches, illustrating consistent fixation patterns and dwell times, with the central thoracic regions receiving the greatest attention. **b** Similarity of fixation trajectories quantified with Pearson correlation for the horizontal and vertical coordinates, indicating moderate spatial agreement. **c** Total time spent per case across a 4 × 4 grid of patches, stratified by representative case distributions across clinical categories. **d** Case-level dispersion of fixation coordinates along both axes, revealing comparable spatial variability when attention is directed to similar anatomical regions.

As shown in Fig. 3, the processed gaze distributions reveal clear and repeatable attention patterns. Each image is divided into a 4 × 4 grid of equal-sized patches, and we calculate the cumulative gaze time within each patch between radiologists. Analysis for 6 × 6 and 8 × 8 grids are shown in Supplementary Fig. 1. Patches in the central thoracic region, which correspond to the lungs and heart (patches 6, 7, 10, and 11) in 4 × 4 grid setting, consistently receive the longest dwell times, while peripheral areas are examined less (Fig. 3a). As shown in Fig. 3c and Supplementary Fig. 1, comparing the viewing patterns of different radiologists across cases grouped by disease category reveals a strong correlation in how their gaze is dispersed. This concentration of attention reflects established diagnostic practice. The lungs and heart are the primary areas where radiologists expect to identify clinically significant findings, including opacities, effusions, cardiomegaly, and vascular changes^34^. We also analyzed clinical categories encompassing both disease findings and support devices, with gazing time recorded using the same patch-based system shown in Fig. 3c. For example, when assessing lung opacity, radiologists examined both lungs across the parenchyma, whereas for support devices, attention was scattered on the peripheral regions. Case-level dispersion plots further confirm shared temporal dynamics in the scanning behavior (Fig. 3d). These findings show that radiologists use consistent patterns when viewing images, following routines from their clinical training. The data reveals valuable insights for developing AI models that can inspect X-rays like radiologists.

### GazeX reproduces radiologist inspection patterns

After pretraining, GazeX can reproduce radiologists’ inspection trajectories, attend to salient gaze clusters, and generate findings informed by expert reading behavior. We evaluate the ability in this section using the test set of REFLACX.

**Fig. 4:**
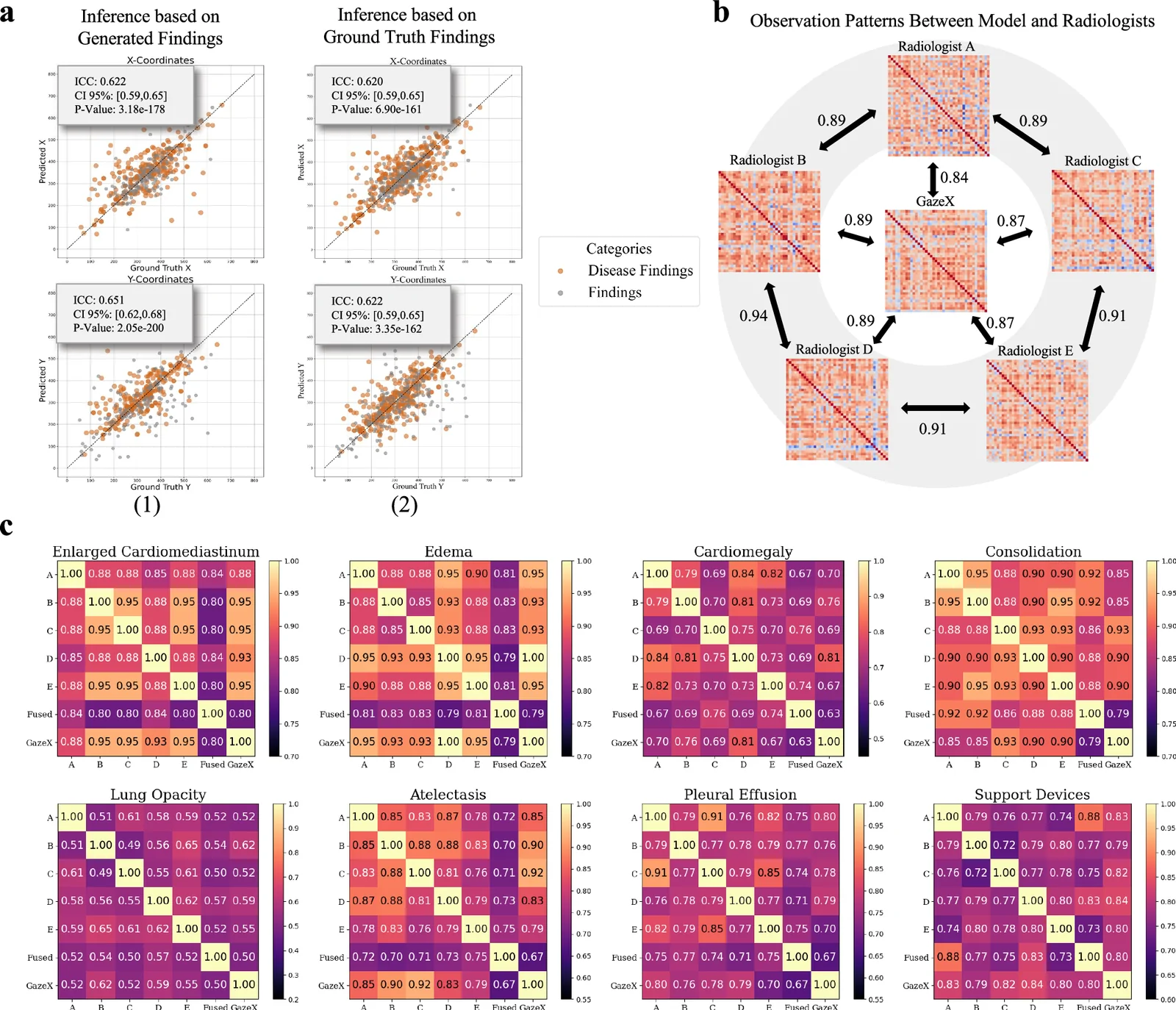
GazeX emulates radiologists’ inspection patterns across spatial, temporal, and disease-specific contexts. **a** Scatter plots showing agreement between GazeX-predicted gaze centroids and those from radiologists, quantified by intraclass correlation coefficients (ICC) for X and Y coordinates under two evaluation settings. **b** Pairwise cosine similarity of attention maps derived from gaze clusters, comparing GazeX with each radiologist and inter-radiologist pairs. High similarity values indicate that the model replicates individual viewing behaviors with fidelity comparable to expert-expert agreement. **c** Disease-specific similarity analysis across the 14 CheXbert categories. The fused category heatmap was computed by averaging the heatmaps from five radiologists. For most conditions, GazeX achieves correlation levels on par with, or approaching, the highest inter-radiologist similarity and remains consistent with aggregated radiologist patterns.

In the first setting (Fig. 4a(1)), GazeX generates both the findings and their associated gaze centroids, simulating a realistic deployment scenario. CheXbert is applied to match generated and reference findings within the same diagnostic category, forming comparable pairs. Since the number of generated findings may differ from the reference findings, the corresponding gaze points may also vary in number. To address this, we used Dynamic Time Warping to align centroid sequences while preserving their temporal order for ICC analysis.

In the second setting (Fig. 4a(2)), ground-truth findings are provided as input to guide centroid extraction, ensuring that spatial comparisons are performed on identical findings. For each disease category in the above two settings, a separate scatter plot is provided in Supplementary Figs. 3 and 4. In both settings, GazeX and radiologists are treated as fixed raters, and their spatial agreement is quantified using ICC(3,1), a two-way mixed effects model with absolute agreement. The results indicate moderate spatial agreement under both conditions: for centroids derived from generated findings, ICC_X = 0.622 and ICC_Y = 0.651; for centroids derived from ground-truth findings, ICC_X = 0.620 and ICC_Y = 0.622. These values suggest that GazeX achieves a spatial alignment level with radiologists that is comparable to agreement levels commonly reported in human-human reliability studies, reflecting a meaningful overlap in fixation patterns.

Inspired by the Representational Similarity Analysis described in^35,36^, we evaluated the similarity of overall viewing patterns by transforming centroids and bounding boxes from both GazeX and radiologists into anisotropic Gaussian attention maps. Each Gaussian was centered at its corresponding centroid, with variance determined by the dimensions of the bounding box. Rather than generating heatmaps directly from the raw radiologists’ gaze data, we employed this approach to mitigate noise inherent in eye-tracking. The centroid and bounding box were derived using a weighted averaging procedure, as described in Methods (Data preparation), thereby capturing the region of highest visual concentration. For each case, we calculated the cosine similarity between the attention maps, generating heatmaps that capture individual inspection styles. As illustrated in Fig. 4b, inter-radiologist inspection behaviors exhibit remarkable consistency, with average similarity scores exceeding 90%. GazeX achieves an average score of 0.872, indicating that the model does not strongly replicate or rely on any single pattern during training. These behavioral patterns remain consistent when subjected to disease-based analysis, suggesting that the observed similarities are not confined to general inspection behaviors but extend to pathology-specific examination strategies.

Following the similar approaches, we conducted disease-specific analysis across the 14 CheXbert categories. From Fig. 4c, we selected eight categories with comparatively representative sample sizes for analysis. The analysis further shows that, for most conditions, GazeX maintains correlation patterns with radiologists that are consistent with expert inspection strategies. Across disease categories, GazeX demonstrated consistent and stable alignment with the fused radiologist heatmaps. For instance, on “Enlarged Cardiomediastinum" and “Edema", GazeX achieved similarity scores of 0.80 and 0.79 to the aggregated radiologist heatmaps, respectively, indicating a high degree of overlap in the regions attended to when diagnosing these conditions. However, in cases with varied inspection patterns, like ’Lung Opacity,’ our model may not identify strong commonalities in observation patterns, yet still demonstrates generalizability across radiologists.

**Fig. 5:**
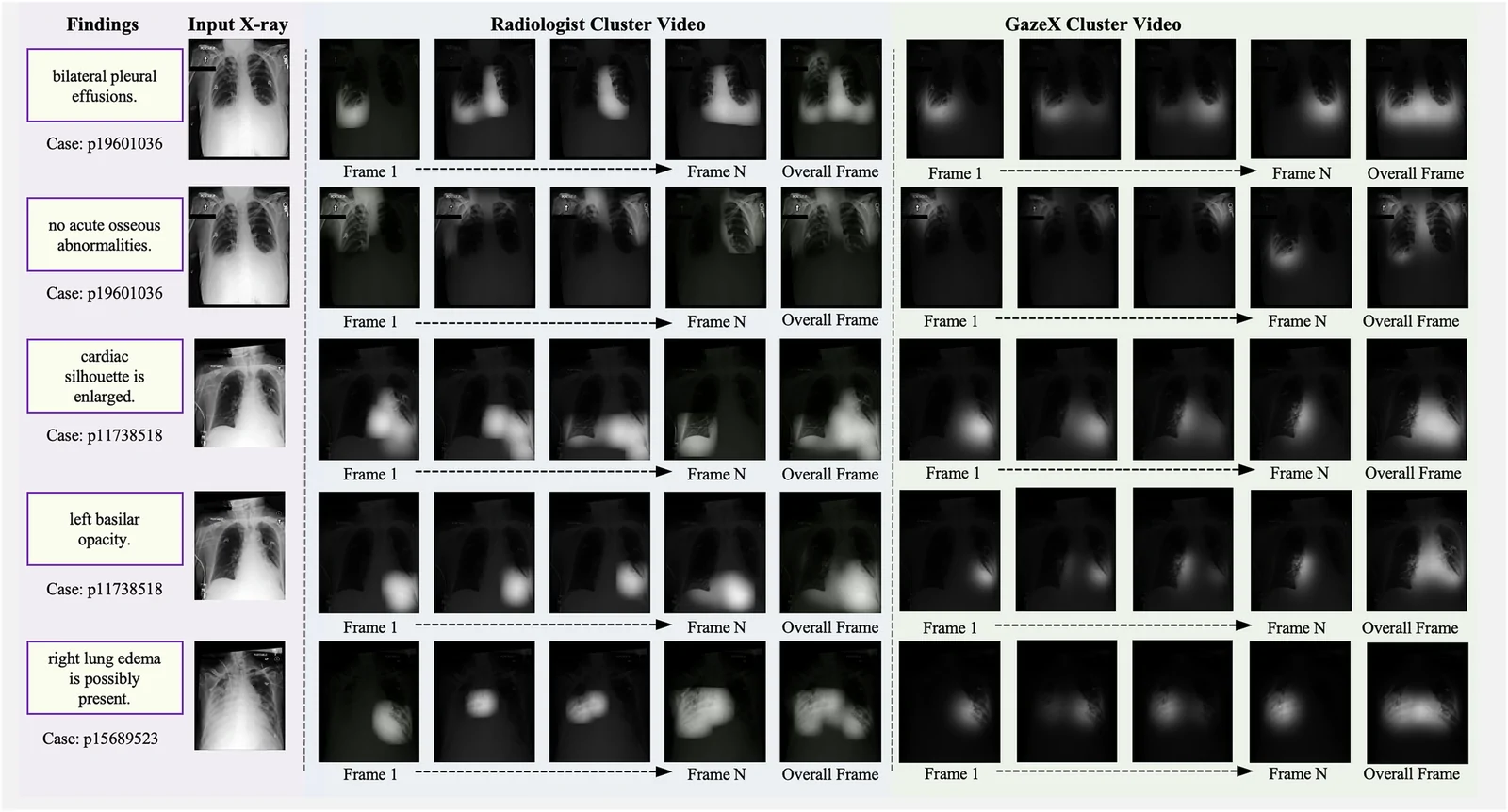
Case-level comparison of disease-specific inspection patterns between GazeX and radiologists. Randomly selected representative examples from five CheXbert disease categories showing model-generated inspection sequences (right) alongside corresponding radiologists' inspection patterns (middle).

Representative examples from five disease categories are presented in Fig. 5. In each example, GazeX’s predicted gaze clusters closely match the spatial distribution and temporal progression of radiologists’ gaze patterns for the same clinical finding. For instance, when examining bilateral pleural effusions, both radiologists and GazeX concentrate their attention on the lung bases while comparing bilateral regions. In cases of ’left basilar opacity,’ the model follows a similar pattern to radiologists by first focusing on the abnormal left basilar area, then examining the corresponding right-sided region to identify potential abnormalities, illustrating a systematic comparative methodology. These qualitative results complement the quantitative correlations, providing visual confirmation that GazeX can emulate radiologist-like inspection behavior in both spatial targeting and temporal sequencing. Representative disease-specific gaze-guided attention videos are shown in Supplementary Figs. 5–16.

### Contribution of inspection pattern modeling

To evaluate how each component contributes to the model’s inspection capability, we tested GazeX with various ablated modules in both zero-shot and LoRA fine-tuned settings, assessing diagnostic accuracy (MIMIC-CXR reports) and attention alignment (REFLACX gaze data).

**Fig. 6:**
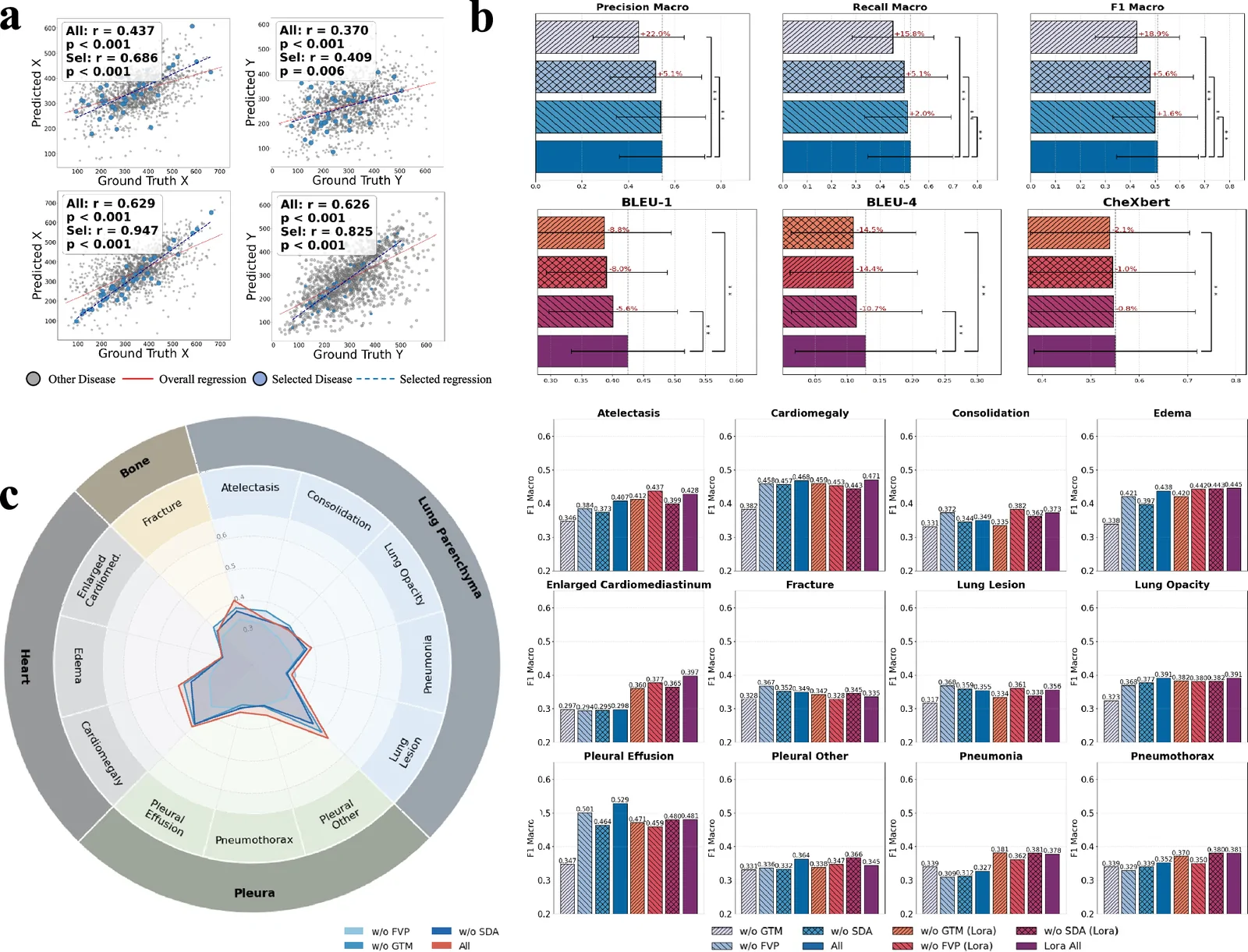
Quantitative evaluation of GazeX against baseline and ablated variants. **a** Correlation between predicted and ground-truth gaze centroids for GazeX and the baseline Qwen2-VL without eye-tracking guided pretrain, reported as Pearson correlation coefficients (r) with corresponding p-values. Results are shown for all disease categories combined and for selected clinically significant categories. **b** Ablation study results. The top row reports classification metrics (Precision, Recall, and F1) for GazeX without LoRA adaptation. The bottom row reports report-generation metrics (BLEU-1, BLEU-4) and clinical correctness (CheXbert F1) for fine-tuned GazeX with LoRA adaptation, under progressive removal of core components (FVP, GTM, and SDA). **c** Condition-specific ablation results for 12 representative medical categories spanning lung, pleura, heart/mediastinum, bone/chest wall, and device-related findings. The radial plot summarizes the overall relative performance of GazeX before downstream task adaptation across disease categories, excluding Support Devices. Bars indicate the comparison between all ablated modules for GazeX and LoRA fine-tuned GazeX for the report-generation task.

We first compare GazeX with baseline, Qwen2-VL, to verify whether radiologist inspection patterns are effectively learned and exploited. In fairness, both models are provided with ground-truth findings to generate corresponding attention regions. Since Qwen2-VL lacks native sentence-level localization, we fine-tuned it using bounding boxes derived from radiologist gaze clusters, mirroring our Fine-Grained Visual Perception module. On the REFLACX test set (Fig. 6a), GazeX achieved substantially higher correlation with radiologist gaze clusters (*r*_*X*_ = 0.629, *r*_*Y*_ = 0.626) than Qwen2-VL (*r*_*X*_ = 0.437, *r*_*Y*_ = 0.370). Gains are especially pronounced in clinically important categories such as support devices, lung lesion, enlarged cardiomediastinum, pleural other, fracture, and pneumothorax (GazeX: *r*_*X*_ = 0.947, *r*_*Y*_ = 0.825; Qwen2-VL: *r*_*X*_ = 0.686, *r*_*Y*_ = 0.409).

We then perform an ablation study to quantify the contribution of the Fine-Grained Visual Perception (FVP), Gaze Trajectory Mimicking (GTM), and Sequential Dependency Awareness (SDA) modules (Fig. 6b). Removing any component reduces classification metrics (Precision, Recall, F1), and even without task-specific fine-tuning, the full GazeX model which driven only by learned inspection patterns, demonstrates strong diagnostic understanding and consistently outperformed all ablated variants. Incorporating LoRA adaptation^37^ for these modules further boosts report-generation metrics (BLEU-1, BLEU-4) and clinical correctness (CheXbert F1). The largest performance drops occur when GTM or SDA are removed, confirming their central role in gaze-informed reasoning. Wilcoxon signed-rank tests show that integrating all three modules yields statistically significant gains (*p* < 0.01).

Condition-specific analysis demonstrates that GazeX maintains strong performance across the 14 CheXbert diagnostic categories (Fig. 6c). The radial plot summarizes relative performance for 13 categories, excluding No Findings, while the accompanying bar charts report CheXbert F1 scores for 12 categories, excluding No Findings and Support Devices. Even without task-specific fine-tuning, GazeX matches or surpasses fine-tuned baselines in several clinically important conditions, with particularly notable gains in lung opacity and pleural effusion. In most categories, both the full GazeX model and its LoRA-adapted variant outperform the ablated configurations, indicating that inspection pattern modeling contributes not only to improved overall accuracy but also to consistent robustness across diverse disease types.

### GazeX augments radiologist findings through collective inspection patterns

**Fig. 7:**
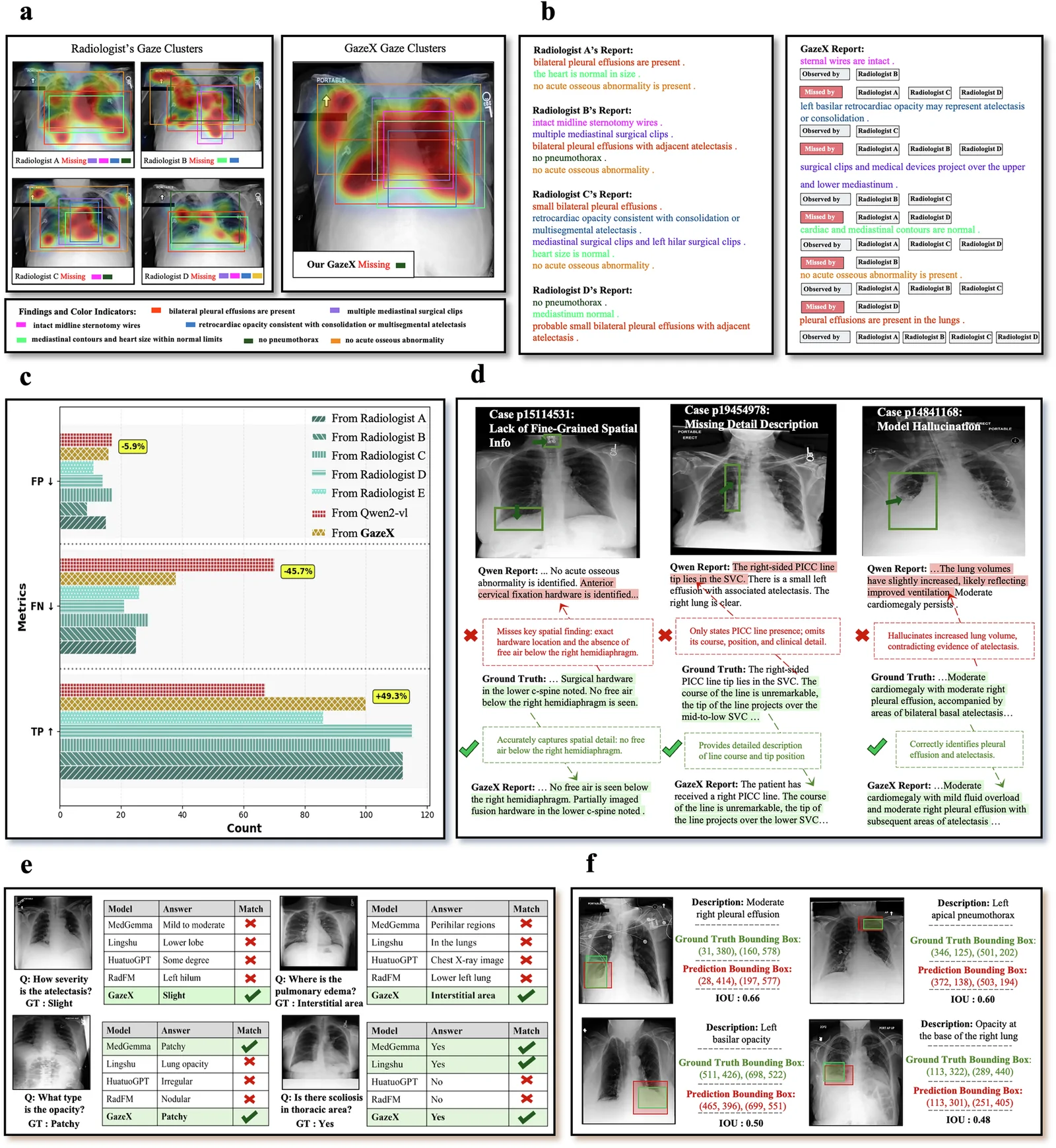
Case study analysis demonstrating the advantages of inspection-guided modeling in GazeX. **a** Comparison of radiologists' gaze clusters and GazeX-predicted gaze clusters on the same case. Colored bounding boxes indicate clinically relevant findings, with “Missing” labels marking unobserved findings. **b** Alignment between GazeX-generated reports and individual radiologists' reports. Colored highlights indicate matched findings; “Observed by” and “Missed by” tags quantify agreement with each radiologist. **c** Quantitative comparison of false positives (FP), false negatives (FN), and true positives (TP) for GazeX, Qwen2-VL baseline, and individual radiologists, using majority-vote consensus reports as reference. **d** Qualitative error analysis on selected cases comparing baseline and GazeX reports. **e** Visual question answering comparisons across multiple medical vision-language models. **f** Localization performance for descriptive findings, with predicted bounding boxes and Intersection-over-Union (IoU) values against ground-truth annotations. The cases selected are random unless specified.

GazeX can potentially identify clinically relevant findings that individual radiologists may not mention. As illustrated in Fig. 7a, GazeX generates findings and highlights regions of interest based on the collective inspection patterns learned from all radiologists. This enables GazeX to detect abnormalities that some individual experts omit, while also aligning its attention with regions commonly examined by radiologists (Fig. 7b). For example, in a case where only Radiologist C reported retrocardiac opacity, GazeX identifies the same region by focusing on areas most relevant to the viewing pattern of Radiologist C. To further evaluate the extent to which GazeX makes diagnostic errors relative to human experts, we compared its outputs with those of individual radiologists and the baseline model, using a majority vote over the radiologists’ report descriptions as the reference standard (Fig. 7c). The results show that GazeX performs on par with the average participating radiologist. GazeX achieves performance comparable to the average behavior of radiologists in identifying findings across different cases, with 100 true positive counts, on par with the radiologists’ average of 105. Specifically, we report the False Negative Rate (FNR), the proportion of truly positive cases that are missed. GazeX achieves an FNR of 27%, comparable to the radiologists’ average FNR of 20%. These findings are consistent with our design objective of providing an explainable model whose outputs can serve as a reliable diagnostic reference for radiologists and clinicians.

### Enhancing diagnostic detail and spatial reasoning with inspection-guided modeling

As shown in Fig. 7d, GazeX generates radiology reports that are more precise and grounded in image evidence compared to the baseline. By incorporating radiologists’ gaze areas paired with their descriptive reports, GazeX integrates fine-grained spatial information and mitigates common errors such as missing detail descriptions or hallucinated findings.

In case p15114531, the baseline Qwen2-VL mentions only anterior fixation hardware, omitting spatial context. GazeX, in contrast, produces a description consistent with the ground truth by recognizing surgical hardware in the lower cervical spine and confirming the absence of free air below the right hemidiaphragm. In case p1945978, Qwen2-VL merely states the presence of a PICC line, whereas GazeX provides a more complete description aligned with the ground truth, detailing its course and tip position. In case p14841168, Qwen2-VL hallucinates an increase in lung volume, contradicting radiological evidence of atelectasis. GazeX avoids this error and produces a report consistent with the ground truth, correctly identifying pleural effusion and atelectasis.

In contrast, the baseline model trained only on paired image-text data often emphasizes lexical similarity rather than spatial grounding, which leads to vague or incomplete descriptions. Even when constrained to designated regions of interest, the baseline may overlook findings that do not consistently manifest within those predefined areas. By integrating inspection-guided supervision, GazeX strengthens the connection between textual descriptions and actual visual evidence, enabling outputs that are more observation-driven and clinically reliable.

Figure 7e, f further illustrate GazeX’s advantage in visual question answering and visual grounding tasks. In clinical question answering (Fig. 7e), GazeX consistently matches ground-truth answers where other models fail, accurately capturing subtle distinctions in abnormality type, severity, and location. In phrase grounding evaluation (Fig. 7f), GazeX demonstrates precise bounding-box predictions that closely align with annotated ground truth, achieving high IoU scores. These results confirm that inspection pattern modeling not only improves narrative report generation, but also enhances spatial reasoning and targeted retrieval of image evidence.

By integrating gaze-derived spatial and temporal information into the pretraining phrase, GazeX aligns its viewing behavior with expert readers and leverages this alignment to improve downstream diagnostic accuracy, enrich spatial detail, and reduce clinically relevant errors. Importantly, this inspection-guided design allows model outputs to be examined in relation to familiar reading steps and anatomical regions, enabling clinicians to verify findings, assess coverage of critical areas, and identify potential omissions during routine review. In this way, GazeX may assist in complementing radiologists by highlighting clinically relevant findings that may be overlooked while maintaining compatibility with established reporting workflows. These results illustrate how GazeX may bridge perceptual and interpretive processes, enabling AI systems to operate in a manner consistent with expert clinical reasoning, thereby improving communication and collaboration between AI systems and clinicians.

### Clinical utility and pathway to uptake

GazeX is designed for clinician-in-the-loop use rather than autonomous diagnosis or reporting. In current clinical practice, fully autonomous chest X-ray reporting remains uncommon, with most approved systems functioning as assistive or triage tools. Consistent with this observation, the inspection behavior learned by GazeX closely aligns with expert clinical practice and achieves performance comparable to inter-radiologist variability. Quantitative analysis shows that the agreement between GazeX and radiologists in spatial and temporal inspection patterns falls within the range observed among experts themselves (Figs. 3 and 4), supporting a deployment paradigm in which model outputs are reviewed and verified by clinicians rather than acted upon autonomously.

A central practical contribution of GazeX is its enforcement of systematic image inspection. Radiologists routinely follow structured reading protocols such as ABCDEF when interpreting chest X-rays, but they do not always explicitly report all normal or negative findings. GazeX models inspection trajectories directly, promoting comprehensive coverage of clinically relevant regions. It also produces structured visual evidence that can be audited step by step during clinical review. As shown in Fig. 3a,c, radiologists exhibit consistent spatial coverage patterns across key thoracic regions. Figure 4a, b further shows that GazeX reproduces these spatial and temporal inspection strategies with fidelity comparable to expert-expert agreement. Together, these properties support coverage assurance, structured verification of model reasoning, and improved trust calibration, enabling clinicians to assess whether conclusions are grounded in appropriate visual evidence.

These inspection-guided behaviors translate into concrete practical outcomes. Case-level analysis indicates that GazeX reduces clinically relevant failure modes observed in baseline vision–language models, including hallucinated findings and omissions (Fig. 7c, d). Moreover, by producing finding-linked localized regions, GazeX enables actionable communication of results. Quantitative grounding improvements (Fig. 2c) and qualitative examples (Fig. 7f) demonstrate that the model can directly answer clinically meaningful questions such as lesion location, supporting clinician-AI communication during verification and clinician-clinician communication by making lesion location easy to reference.

In practical use, GazeX may serve as an assistive verification tool in situations where radiologists remain uncertain after an initial interpretation. Notably, given the modest model size of 2 billion parameters, deployment is feasible on reasonably priced GPUs, making the system accessible for routine clinical environments without prohibitive hardware costs. By highlighting image regions associated with diagnostic ambiguity, GazeX, having learned from the visual inspection patterns of multiple experts and emulating how other radiologists would assess these regions in an X-ray, provides a reference for second-pass review. This enables clinicians to revisit specific anatomical areas during follow-up inspection and cross-checking, while preserving their clinical judgment. GazeX is intended as a decision-support tool that proposes findings accompanied by linked visual evidence, enabling clinician verification and editing rather than serving as a stand-alone diagnostic model that generates final reports. We frame the contribution of GazeX in terms of transparency, trustworthiness, and support for human verification: by surfacing inspection-aligned evidence for each finding, the model offers an interpretable interface that allows clinicians to scrutinize, validate, or revise its outputs.

### Limitations and future directions

The REFLACX dataset provides board-certified, subspecialty-trained thoracic radiologists’ viewing patterns under controlled conditions, from which our model benefits by learning gaze behaviors in a clean and consistent manner. However, it does not fully reflect real-world clinical practice, where radiologists routinely compare the current X-ray with prior studies across multiple screens to assess disease progression, a behavior that cannot be captured from the present data. In addition, as demonstrated in^38,39^, radiologist fatigue, whether oculomotor, cognitive, or shift-induced, produces objective, quantifiable changes in eye-movement behavior that translate into measurable declines in diagnostic performance. However, no quantitative fatigue measures (e.g., Swedish Occupational Fatigue Inventory scores, shift status, or time-on-task indicators) are provided in our training dataset. Consequently, we are unable to estimate how fatigue may have influenced the recorded gaze patterns across radiologists. However, to minimize the potential influence of fatigue-related artifacts, we applied a preprocessing step to the gaze data: fixation points falling outside the image boundaries, as well as sudden, erratic shifts occurring during sustained focus on a single region, were filtered out. This ensured that only high-quality gaze samples, those most reliably associated with disease findings, were retained for training. Other than that, the dataset contains only frontal-view X-rays, which may limit the model’s ability to handle findings that require joint reasoning over frontal and lateral views; in particular, the model cannot learn the decision process by which a radiologist elects to consult the lateral view when the frontal view alone is inconclusive. Finally, although the dataset records zoom-in and enlargement operations performed during detailed inspection of specific regions, our current data processing does not fully model this behavior: we assign higher attention weights to the focused areas but do not explicitly inform the model that the radiologist intentionally zoomed in. Future studies will incorporate larger and more diverse radiologist gaze datasets that better generalize to real clinical behaviors, thereby improving both the reasoning process and the quality of the model’s output.

Building on these advances, future work will focus on evaluating the practical impact of inspection-guided evidence in clinical use. First, although GazeX is designed to support systematic image review, its effects on reporting efficiency, miss rates, and false-positive rates have not yet been quantified and will need to be assessed in prospective reader studies. Future studies will also examine trust calibration, including how clinician confidence aligns with case difficulty when inspection cues are provided, to better understand how such evidence supports human verification in practice, especially for the cases mentioned in Supplementary Fig. 17. In addition, our evaluation dataset does not include rare disease cases, which may limit robustness when encountering uncommon conditions in real-world practice. Expanding the dataset to cover a broader spectrum of pathologies represents an important direction for improving generalization. The current framework does not incorporate external medical knowledge sources, such as retrieval-augmented generation (RAG), which could provide additional clinical context to guide inspection and interpretation. Finally, this study is restricted to chest X-rays, and the GazeX architecture has not yet been adapted or validated for other imaging modalities such as CT, MRI, or ultrasound. Future work will explore integrating domain knowledge in an auditable manner and extending the inspection-guided framework to additional modalities.

## Methods

### Dataset for pretraining

The REFLACX dataset contains 3032 synchronized eye-tracking and report transcription cases, for a total of 2616 unique frontal chest X-ray images from the MIMIC-CXR dataset, each read by one or more of five board-certified radiologists. During each case, radiologists’ descriptive findings and eye movements were recorded while they viewed and dictated X-ray interpretations, with eye tracking captured continuously at 1000 Hz. Data were collected in three phases. Phases 1 and 2 involve multiple radiologists viewing the same X-rays. Phase 1 used 59 chest X-rays, and Phase 2 used 50 chest X-rays. Each X-ray was interpreted by the same group of radiologists (up to 5). This produced 285 and 240 eye-tracking recordings for Phases 1 and 2, respectively, with some recordings being dropped due to technical issues. Phase 3 represents the main component of the dataset, including 2507 unique cases, and each radiologist reviewed a different set of images. For training and evaluation, we adopt an 80/20 split over the full set of 3032 recordings. Specifically, we use all 240 cases from Phase 2 and randomly sample 350 from Phase 3 and Phase 1 to form a 590-case test set (~20%), while the remaining 2442 cases are used for training.

### Data preparation

**Fig. 8:**
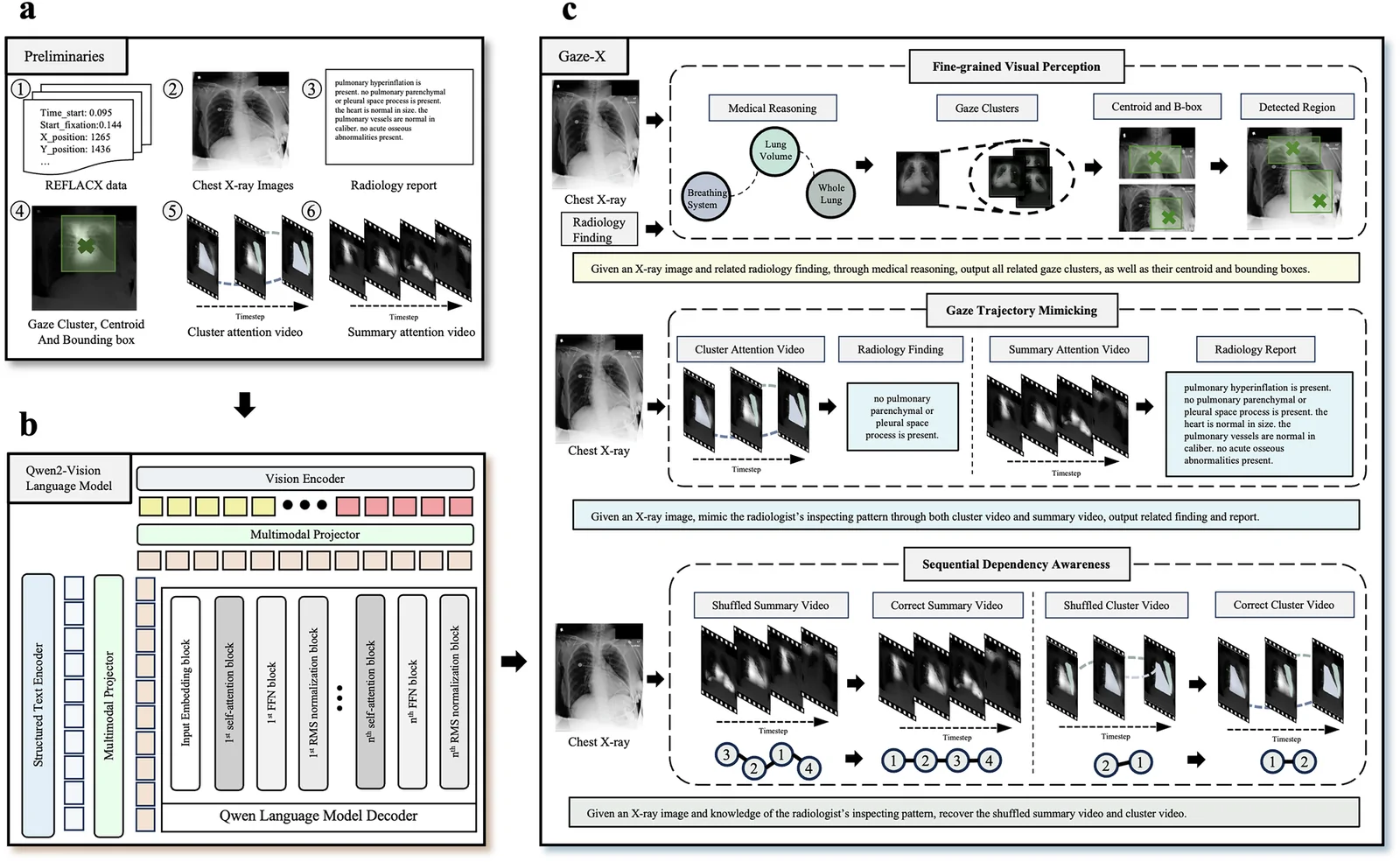
Overview of the GazeX framework and its components. **a** Preliminaries: The REFLACX dataset provides synchronized radiology reports, frontal chest X-ray images, and eye-tracking data. Gaze clusters are extracted from fixation points, with each cluster characterized by its centroid and bounding box. These are assembled into cluster attention videos (per finding) and a summary attention video (aggregating all findings). **b** GazeX architecture: Built on the Qwen2-Vision-Language backbone with optimized structured text encoder, vision encoder, and multimodal projector. **c** Core modules: Fine-grained Visual Perception (FVP): Maps textual findings to gaze clusters, centroids, and bounding boxes via medical reasoning, enabling local-level spatial grounding. Gaze Trajectory Mimicking (GTM): Trains the model to follow radiologist-like inspection patterns using both cluster attention videos and summary attention videos. Sequential Dependency Awareness (SDA): Recovers the correct temporal order of shuffled attention videos, fostering global-level interpretive reasoning.

**Fig. 9:**
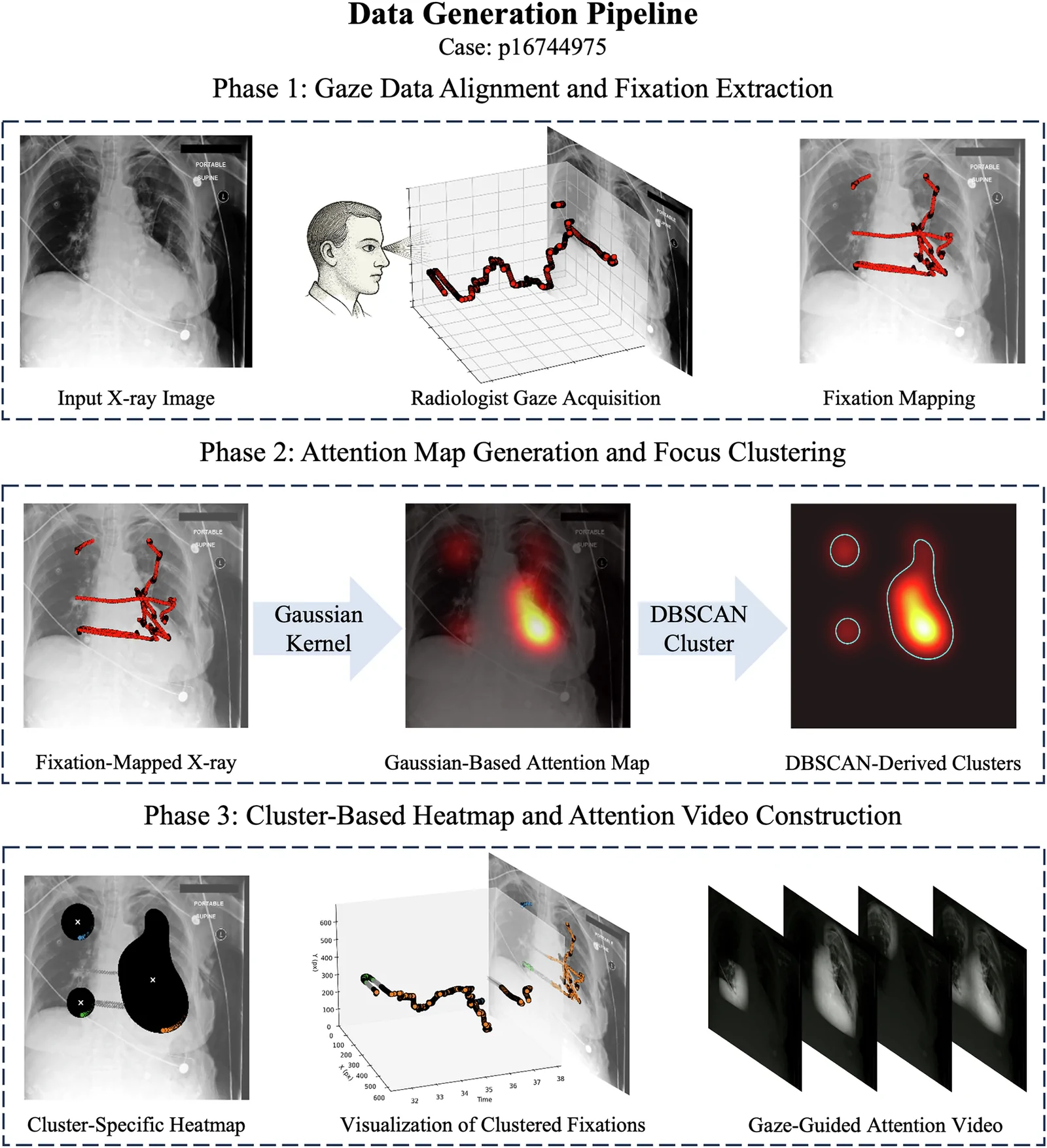
Illustrative example of the data processing pipeline. Given a chest X-ray image, following the pipeline defined by the pseudocode Algorithm 1 in supplementary data, gaze-guided attention videos are generated through three phases for model training. Phase 1: Acquire radiologist gaze records and extract fixations based on their verbal descriptions of the regions of interest. Phase 2: For each specific verbal description, apply Gaussian attention map to the corresponding fixation data, followed by DBSCAN clustering to identify attention clusters. Phase 3: Refine the extracted attention clusters and construct the final gaze-guided attention videos for use in model training.

All chest X-ray images in REFLACX are downsampled by a factor of 4 along both spatial dimensions (that is, width and height), resulting in a total area scaling of 1/16. The gaze coordinates are correspondingly scaled by a factor of 4 to remain aligned with the resized images. For each verbal mention of an abnormality, we extract the set of fixation points *F*_*i*_ = (*x*_*i*_,*y*_*i*_,*t*_*i*_) occurring within a 2.5-second window prior to the timestamp of the mention. As reported in^40^, the dataset analyzes the temporal interval between when a radiologist visually examines a region and when the corresponding verbal diagnosis is articulated, revealing an approximately 2.5-second window between fixation and verbalization. Accordingly, we treat this interval as the temporal sequence over which the radiologist inspects the image and subsequently associates the visual evidence with the diagnostic finding. Each fixation point is used to generate a 2D Gaussian heatmap *G* centered at (*x*_*i*_, *y*_*i*_) with standard deviations *σ*_*x*_ and *σ*_*y*_ along the horizontal and vertical axes, respectively. The values of *σ*_*x*_ and *σ*_*y*_ are derived from the angular resolution of the image at the fixation point, expressed in pixels per degree of visual angle (that is, angular resolution x pixels per degree and angular resolution y pixels per degree). Each Gaussian is evaluated over a bounded window with radius 3 times of $\max ({\sigma }_{x},{\sigma }_{y})$, which captures more than 99% of the Gaussian mass. The resulting Gaussians are summed to produce a temporally aligned spatial attention map for each finding. We then apply DBSCAN clustering^41^ (*ϵ* = 0.3, MinPts = 10) to the resulting attention maps to isolate distinct regions of visual focus. Here, *ϵ* defines the maximum distance between points to be considered neighbors, and MinPts is the minimum number of points required to form a dense region, allowing the algorithm to identify focused areas while filtering out sparse noise. Given that the eye-tracking device in the dataset records gaze at 1000 Hz, the threshold of MinPts remains highly sensitive and is capable of capturing even rapid peripheral fixations. To validate this choice, we additionally evaluated minimum-point thresholds of 1, 5, and 20, all of which produced clusters identical to those obtained with a threshold of 10. For each cluster, we apply the previously described method to the corresponding coordinates in order to generate a new heatmap. The centroid of each cluster is subsequently computed via intensity-weighted averaging of the corresponding coordinates, while bounding box anchors are derived from the maximum and minimum XY coordinates of the top 20% highest-intensity pixels within the heatmap. These clusters are used to construct an attention video in which the clusters are sequentially overlapped with the resized chest X-ray. Each finding with *K* clusters contributes *K* + 1 frames to their attention video *V*_*m*_, one for each cluster in temporal order and one summary frame *H*_*total*_ showing all clusters for that finding. Suppose a report contains *M* findings, with each finding *m* associated with video *V*_*m*_ containing *K* + 1 frames. Then the total number of frames in the summary attention video *V* is given by $\mathop{\sum }\nolimits_{i = 1}^{| M| }({K}_{i}+1)$. An illustration of the REFLACX data, chest X-ray image, radiology report, gaze group, cluster’s centroid *c* and bounding box *b*, cluster video *V*_*m*_ of a description and summary video *V* of the entire diagnosis on a chest X-ray image is provided in Fig. 8a and the pipeline for generating attention videos is illustrated in Fig. 9. Unless otherwise specified, we assume that the dataset used for evaluating inter-radiologist consensus was collected sequentially, referring to the code provided by the dataset for inter-rater analysis.

### GazeX model architecture

We propose GazeX (Fig. 8), a 2 billion-parameter foundation model for chest X-rays that builds on the Qwen2-Vision-Language architecture, with optimized structured text encoder, vision encoder, and multimodal projector components. To enable spatially grounded and context-aware understanding, GazeX integrates three modules to capture different levels of visual and semantic complexity: fine-grained visual perception (FVP), gaze trajectory mimicking (GTM), and sequential dependency awareness (SDA).

In FVP, we construct a medical reasoning dataset that pairs each radiology finding with anatomical locations, which are further refined into gaze-based clusters. The model is trained to predict these clusters along with their centroids and bounding boxes, allowing it to establish precise spatial correspondences between textual findings and visual evidence in the X-ray. GTM enables GazeX to learn radiologist-like inspection patterns by supervising it with cluster attention videos *v*_*i*_ aligned with specific findings and summary attention videos *V* aligned with complete radiology reports. By modeling not only where radiologists look but also how their attention unfolds over time, GTM exposes the model to the cognitive trajectory of expert visual interpretation, an implicit chain of perceptual evidence that may mirror the reasoning process underlying diagnostic conclusions. This guides the model to attend to both localized and holistic regions of the chest X-ray in a manner consistent with clinical reading behavior. Finally, in SDA, we shuffle the temporal order of both *v*_*i*_ and *V* and train GazeX to recover the correct viewing sequence, promoting sensitivity to the temporal structure of radiological workflows and enhancing global contextual understanding. Details of the data processing pipeline are provided in Supplementary Algorithm 1.

### Downstream evaluation tasks

To assess the effectiveness of GazeX across different levels of visual-linguistic understanding, we evaluate its performance on three downstream tasks of decreasing complexity: radiology report generation, visual question answering and visual grounding. These tasks are designed to investigate complementary capabilities of the model, ranging from language generation to contextual interpretation and spatial localization. Report-generation evaluates global-level understanding by requiring the model to synthesize coherent radiological summaries that integrate information throughout the entire X-ray image. Visual question answering assesses local-level comprehension by testing the model’s ability to answer fine-grained clinical questions about visual content. Visual grounding targets image-level understanding, which requires aligning free-text phrases with specific anatomical regions. For all datasets, we adhere to their official training, validation, and test splits. All chest X-ray images and the corresponding bounding box coordinates are downsampled by a factor of 4 along both spatial dimensions (that is, width and height), corresponding to a 1/16 reduction in spatial area.

### Datasets used for radiology report generation

We use two widely adopted datasets: MIMIC-CXR^42^ and IU X-Ray^43^. MIMIC-CXR contains 377,110 chest X-ray images paired with 227,835 free-text radiology reports, while IU X-Ray includes 7470 images and 3,955 reports. For both cases, we target the “Findings" section of each report for generation. We selected representative chest X-ray report-generation models for this evaluation, including R2Gen^21^, CMN^22^, MA^1^, RGRG^25^, R2GenGPT^44^, PromptMRG^45^, STREAM^26^, and our proposed GazeX.

### Datasets used for visual question answering

We use the Medical-CXR-VQA dataset^29^, which includes 780,014 question-answer pairs based on MIMIC-CXR images. The questions span six categories: abnormality (190,525 pairs), location (104,680 pairs), type (69,486 pairs), severity level (111,715 pairs), view (92,048 pairs) and presence (211,560 pairs), supporting both visual and linguistic reasoning. We selected both models fine-tuned on the Medical-CXR-VQA dataset and zero-shot foundation models, including LLaVA-Med-7B^46^, RadFM-14B^47^, HuatuoGPT-Vision-7B^48^, HuatuoGPT-Vision-32B^48^, Bio-Medical-MultiModal-Llama-8B^49^, Medical-CXR-VQA^29^, MedGemma-4B-it^50^, Lingshu-7B^51^, and Lingshu-32B^51^. We tested model variance across different parameter sizes (in billions) to compare the trade-off between model parameter size and performance, since smaller models can be more feasible for real-world implementation with limited resources.

### Datasets used for visual grounding

We use the MS-CXR dataset^31^, which provides 1,153 image-sentence pairs consisting of bounding boxes and associated phrases derived from MIMIC-CXR. These structured annotations enable precise grounding of visual regions to fine-grained textual descriptions. We selected models with different training methods, including ChEX^32^ (visual grounding foundation model), TransVG^33^ (supervised learning model for visual grounding), BioVIL^31^ (CLIP-based learning model for visual grounding), and OmniFM-DR^30^ (generative visual-grounding model).

### Evaluation metrics for radiology report generation

Performance is assessed using standard natural language generation metrics, including BLEU-1 to BLEU-4^52^, METEOR^53^, and ROUGE-L^54^, to measure textual similarity. In addition, we use the CheXbert^55^ labeler to assign reports to 14 common disease categories to provide an assessment of the clinical accuracy of the generated text. For model comparison with existing approaches, we adopt the mainstream evaluation methodology that employs F1 scores, where only positive findings are classified as positive cases. In the ablation study, we observe that the pretraining dataset contains radiologist-generated findings characterized by inherent uncertainty, which our model subsequently learns to replicate, thereby reflecting the diagnostic uncertainty present in radiological examinations. To comprehensively evaluate this behavior, we implement a multiclass classification framework.

### Evaluation metrics for visual question answering

We report top1 phrase-matching accuracy^29^. Predictions are decomposed into phrases and are considered correct if any predicted phrase exactly matches any ground-truth phrase.

### Evaluation metrics for visual grounding

We evaluate this task using mean Intersection over Union (mIoU) and mean Average Precision (mAP)^31^. For mIoU, predicted bounding boxes are selected based on confidence scores, merged into a binary mask, and compared to a merged ground-truth mask using IoU thresholds of {0.1, 0.2, 0.3, 0.4, and 0.5}. IoU is first averaged across all sentences for each threshold and then averaged across thresholds. For mAP, the task is treated as object detection, with each sentence corresponding to a unique class. The average precision is computed using IoU thresholds of {0.1, 0.2, 0.3, 0.4, 0.5, 0.6, and 0.7} and averaged across all classes and thresholds.

## Supplementary information

Supplementary information

## Data Availability

All datasets used in this study are listed in the Methods section and are publicly available. MIMIC-CXR and IU X-Ray are available at https://physionet.org/content/mimic-cxr/2.1.0/ and https://www.kaggle.com/datasets/raddar/chest-xrays-indiana-university, respectively. The Medical-CXR-VQA dataset is available at https://physionet.org/content/medical-cxr-vqa-dataset/1.0.0/. The MS-CXR dataset and corresponding annotations are available at https://physionet.org/content/ms-cxr/1.1.0/. The REFLACX dataset, including chest X-ray reports and radiologist eye-tracking data, is available at https://physionet.org/content/reflacx-xray-localization/1.0.0/.

## Code availability

The code for preprocessing and reproducing the results is in the GitHub repository: GazeX.
