# Supplementary material for: Seeing through experts' eyes: a foundational vision-language model trained on radiologists' gaze and reasoning
Source: NPJ Artif Intell. 2026 Jul 13;2(1):87. doi: 10.1038/s44387-026-00136-9 (PMC13736516; doi:10.1038/s44387-026-00136-9)
Supplement: Supplementary file 1 [file 44387_2026_136_MOESM1_ESM.pdf]

Supplementary Information for Seeing Through Experts' Eyes: A Foundational Vision-Language Model Trained on Radiologists' Gaze and Reasoning

Kinhei Lee*[1], Peiyuan Jing*[1], Zhenxuan Zhang*†[1], Yue Yang[1], Tao Wang[1,2], Dominic C Marshall[3], Yingying Fang[1], and Guang Yang †[1,4,5,6]

[1]Bioengineering Department and Imperial-X, Imperial College London, London W12 7SL, UK
[2]College of physics and information engineering, Fuzhou University, Fuzhou, 350108, China
[3]Department of Surgery and Cancer, Imperial College London, London W12 0NN, UK
[4]National Heart and Lung Institute, Imperial College London, London SW7 2AZ, UK
[5]Cardiovascular Research Centre, Royal Brompton Hospital, London SW3 6NP, UK
[6]School of Biomedical Engineering & Imaging Sciences, King's College London, London WC2R 2LS, UK

List of Figures

1 Total fixation duration across patches in different grid settings 10
2 Scatter plots showing case-level dispersion of radiologists' fixation coordinates 11
3 Scatter plots showing the agreement between GazeX predicted gaze centroids based on ground truth findings and those from radiologists 12
4 Scatter plots showing the agreement between GazeX predicted gaze centroids and those from radiologists 13
5 Gaze-guided attention videos of different training samples for cardiomegaly 14
6 Gaze-guided attention videos of different training samples for lung opacity 15
7 Gaze-guided attention videos of different training samples for pleural effusion 16
8 Gaze-guided attention videos of different training samples for support device 17
9 Gaze-guided attention videos of different training samples for pneumothorax 18
10 Gaze-guided attention videos of different training samples for lung lesion 19
11 Gaze-guided attention videos of different training samples for atelectasis 20
12 Gaze-guided attention videos of different training samples for consolidation 21
13 Gaze-guided attention videos of different training samples for edema 22
14 Gaze-guided attention videos of different training samples for fracture 23
15 Gaze-guided attention videos of different training samples for enlarged cardiom. 24
16 Gaze-guided attention videos of different training samples for other condition occurred in pleural space 25
17 Suboptimal cases in GazeX examination compared to expert radiologists 26
18 Ablation study on single-reader versus fused multi-reader gaze supervision 27
19 Comparison of different report generation methods across GREEN and RadGraph-F1 28

List of Tables

1 Comparison of different report generation methods across various evaluation metrics in MIMIC-CXR [1] and IU X-ray [2]. 29
2 Comparison of different medical foundation models in X-ray VQA task [3]. 30

*These authors contributed equally to this work.
†Corresponding Author. Email: z.zhenxuan24@imperial.ac.uk, g.yang@imperial.ac.uk

3 Comparison of different visual grounding methods across various evaluation metrics in MS-CXR dataset [4]. 31
4 Legends for the video files provided. 32

A RELATED WORK

A.1 Chest X-ray Image Analysis

Recent multi-modal approaches for chest X-ray interpretation encompass both contrastive representation learning and more advanced vision–language modeling paradigms. Early methods such as ConVIRT [5], CXR-LLaVA [6], CML [7], and BioVIL [4] primarily rely on image–text contrastive alignment to learn joint embeddings. While these contrastive frameworks demonstrate strong downstream performance in classification and report generation, contrastive learning [8] inherently prioritizes global similarity and often lacks fine-grained spatial grounding, limiting the model's ability to capture localized abnormalities [9]. For example, the CXR-LLaVA image encoder [6] is first pre-trained contrastively [10] and subsequently aligned with an LLM (Llama[11]), meaning that any failure to encode subtle disease regions during pre-training propagates through later instruction tuning, leading to weak spatial interpretability and incomplete diagnostic reasoning.

Beyond contrastive-learning approaches, recent LVLM-style multimodal models aim to improve clinical fidelity by integrating richer cross-modal reasoning. Flamingo-CXR [12] adapts a large vision–language model for chest X-ray report generation and clinician-guided evaluation, while RadAlign [13] enhances diagnostic accuracy through vision–language concept alignment and retrieval-based reasoning. Similarly, M4CXR [14] supports multitask chest X-ray interpretation—including report generation, VQA, and visual grounding—within a unified multimodal LLM framework. Meanwhile, grounding-oriented X-ray methods such as MedRPG [15] and MedKLIP [16] introduce phrase-level or knowledge-enhanced spatial alignment to improve disease localization. However, despite these advances, most models still lack structured modeling of radiologists' diagnostic workflow or explicit spatial–temporal reasoning, resulting in limited clinical interpretability and verifiability [17; 18]. These limitations highlight the need for training strategies that incorporate spatially explicit and clinically grounded supervision signals during foundation model development.

A.2 Medical Large-Vision-Language Foundation Model

Foundation models, which are trained on large datasets, have emerged as a transformative approach in deep learning for various applications, for example, diagnostic imaging [19; 20; 21; 22]. Large-Vision-Language Foundation Models (LVLMs) applied in medical field have been developed as unified, end-to-end frameworks, integrating vision and language modules to enhance clinical fidelity in different downstream tasks such as visual question answering, disease classification and localization [6; 23; 24; 25; 26; 27]. RadFM [23], HuatuoGPT-Vision [24], and MedVLM-R1 [25] are advanced medical LVLMs that demonstrate significant performance across various downstream tasks. LVLMs, pretrained on extensive visual and textual paired datasets to enhance alignment and foundational capabilities, can be adapted to various tasks. However, their explainability and spatial verifiability remain limited. For instance, while an LVLM may correctly identify a disease, when prompted to highlight the observed regions, it may fail to accurately delineate the relevant areas, leading to potential hallucinations [28; 29]. For example, although MedVLM-R1 [25] improves diagnostic reasoning through reinforcement learning, it provides limited spatial grounding or alignment with practical diagnostic workflows. Incorporating expert guidance into the training pipeline could align the model's examination process more closely with the practices of radiologists in clinical settings to ensure clinical reliability and interpretability [30; 31]. This approach may address the challenges of limited explainability and spatial verifiability, while also reducing hallucination issues. Eye-tracking data, which captures radiologists' observation and examination patterns, may serve as an ideal resource for training models to emulate expert diagnostic processes.

A.3 Eye-tracking based Method

Given the limited spatial grounding and lack of alignment with expert diagnostic workflows in existing Medical LVLMs, eye-tracking-based methods have emerged as a promising direction to incorporate human visual priors, enabling models to better attend to clinically relevant regions and mimic expert reasoning patterns [32; 33; 34] Especially in radiology, when radiologists examine chest X-ray images, they follow a specific search pattern to identify visual clues, integrating both global and local observations to reach a final diagnosis [33; 35; 36]. Sequential patterns captured in eye-tracking data [37; 38; 37] provide informative reasoning cues that can be extracted and applied as constraints, enabling the model to examine images in a manner similar to that of a radiologist. Recent efforts utilize gaze signals as weak supervision to enhance model robustness under data scarcity. For instance, FocusContrast [39] utilizes gaze-derived attention masks to replace random data augmentations in contrastive learning. While effective, the gaze information is used statically and lacks modeling of diagnostic flow or region

interactions. EGMA [40] aligns radiologist gaze with textual phrases to improve vision-language correspondence. Yet, similar to FocusContrast, it overlooks temporal gaze patterns, thus failing to capture how radiologists progressively reason over findings. Grouped Fusion Networks [41] directly integrate gaze maps into a dual-stream U-Net architecture for mammogram segmentation, showing effectiveness in guiding local predictions. Nonetheless, it remains limited to segmentation tasks and does not generalize across broader vision-language applications.

B Experimental Settings

B.1 Data Processing Pipeline

This section defines the key constructs used throughout our method, including fixations, attention bounding boxes, centroids, attention maps, and the gaze-guided video representation. Our objective is to transform raw gaze data into spatially and temporally structured representations of visual attention that can be integrated into the training process.

Let $I \in \mathbb{R}^{H\times W}$ denote a chest X-ray image, $G = (x_i, y_i, t_i)$ the sequence of time-stamped gaze points recorded during radiologist interpretation, and M the corresponding findings collected during radiologists' examination.

For each finding $m \in M$, a mention interval $[t_m^{\text{start}}, t_m^{\text{end}}]$ is obtained from time-stamped transcriptions. Associated fixation points are defined as:

$$F_m = \left\{(x_j, y_j, t_j) \in G \;\middle|\; t_j \in [t_m^{\text{start}}, t_m^{\text{end}}] \wedge x_j \in [Q_x^{0.05}, Q_x^{0.95}] \wedge y_j \in [Q_y^{0.05}, Q_y^{0.95}]\right\}, \tag{S1}$$

where Q_x and Q_y represents the percentile of X and Y coordinates recorded in the time interval for the finding m.

Each fixation point is treated as centroid to generate a 2D Gaussian heatmap G with standard deviations σ_x and σ_y along the horizontal and vertical axes, respectively. The values of σ_x and σ_y are derived from the angular resolution of the image at the fixation point, expressed in pixels per degree of visual angle (that is, angular resolution x pixels per degree and angular resolution y pixels per degree). An attention map $A_m \in \mathbb{R}^{H\times W}$ is computed by placing a Gaussian kernel on each fixation point (x_j, y_j) from F_m:

$$A_m(x,y) = \sum_{(x_j,y_j)\in F_m} \exp\left(-\frac{(x-x_j)^2}{2\sigma_x^2} - \frac{(y-y_j)^2}{2\sigma_y^2}\right), \tag{S2}$$

where σ_x and σ_y are empirically estimated standard deviations that reflect the spatial spread of vision. Each Gaussian is evaluated over a bounded window with radius 3 times of $\max(\sigma_x, \sigma_y)$, which captures more than 99% of the Gaussian mass. The resulting Gaussians are summed to produce a temporally aligned spatial attention map for each finding.

For each attention map A_m, we perform DBSCAN clustering [42] ($\epsilon = 0.3$, MinPts $= 10$) to isolate distinct regions of visual focus. Here, ϵ defines the maximum distance between points to be considered neighbors, and MinPts is the minimum number of points required to form a dense region, allowing the algorithm to identify focused areas while filtering out sparse noise. One advantage of extracting visual focuses from a whole attention map is that it clearly highlights the important parts for model learning. For example, when diagnosing bilateral pleural effusions, the observation focus of radiologist splits into two parts, the left and right lung bases, for comparison, instead of showing the whole attention map. For each cluster, we apply the previously described method for generating A_m to the corresponding coordinates within the cluster in order to generate a new heatmap H_k. Finally, for each finding m, heatmaps $\{H_k\}_{k=1}^K$ were generated, where K equals the number of clusters. Based on H_k, the bounding box and most intense centroid were extracted for model learning as follows:

$$\mathbf{c}_k = (c_{k,x}, c_{k,y}) = \left(\frac{\sum_{x=1}^{W}\sum_{y=1}^{H} x\cdot H_k(x,y)}{\sum_{x=1}^{W}\sum_{y=1}^{H} H_k(x,y)}, \frac{\sum_{x=1}^{W}\sum_{y=1}^{H} y\cdot H_k(x,y)}{\sum_{x=1}^{W}\sum_{y=1}^{H} H_k(x,y)}\right), \tag{S3}$$

where $\mathbf{c}_k$ represents the intensity-weighted average of the corresponding coordinates. Bounding box anchors were derived from the maximum and minimum XY coordinates of the top 20% highest-intensity pixels within the heatmap.

An attention sequence video V_m for finding m is constructed by overlaying each of $\{H_k\}_{k=1}^K$ onto I: $V_m = \{I \odot H_k\}_{k=1}^K \cup \{H_{\text{total}}\}$ consists of $K+1$ frames, where K denotes the number of clustered focus areas and H_{total} indicates one summary frame showing all clusters, $\sum_{k=1}^K I \odot H_k$, for that finding. For the whole diagnosis process of a chest X-ray image, the final attention sequence video would be $\bigcup_{m=1}^{|M|} V_m$. Detailed in Supplementary Fig. 5

Algorithm 1: DBSCAN and Cluster Heatmap Generation for A_m

Require: A_m: attention map of size $H \times W$, $\epsilon = 0.3$, $MinPts = 10$, $threshold = 0.1$, $\{(x_i, y_i, \sigma_{x_i}, \sigma_{y_i})\}_{i=1}^{N}$: original gaze points

Ensure: $\{H_k\}_{k=1}^{K}$: final heatmaps for each cluster from A_m

Phase 1: Extract High-Intensity Coordinates

Initialize $P \leftarrow \emptyset$

$(y_{coords}, x_{coords}) \leftarrow \text{Where} A_m[y_{coords}, x_{coords}] > threshold$

$I_{coords} \leftarrow A_m[y_{coords}, x_{coords}]$

$P \leftarrow \{(x_j, y_j, I_j)\}_{j=1}^{M}$ where $M = |x_{coords}|$

Phase 2: DBSCAN Clustering

$XY \leftarrow \text{StandardScaler}([x_{coords}, y_{coords}])$ {normalize space}

$D \leftarrow [XY, I_{coords}]$ {3D feature space: $[x\_norm, y\_norm, intensity]$}

$L \leftarrow \text{DBSCAN}(D, \epsilon, MinPts)$ {cluster labels}

$K \leftarrow |\{c \mid c \in \text{unique}(L), c \neq -1\}|$ {non-noise clusters}

Phase 3: Generate Cluster Heatmaps

$\{H_k\}_{k=1}^{K} \leftarrow \emptyset$

for each cluster label $c_k \in \{0, 1, \ldots, K-1\}$ **do**

  $C_k \leftarrow \{(x_j, y_j, I_j) \in P \mid L_j = c_k\}$

  Initialize $H_k \leftarrow \mathbf{0}_{H \times W}$

  for each $(x_j, y_j, I_j) \in C_k$ **do**

    Find corresponding gaze point $(x_i^*, y_i^*, \sigma_{x_i}^*, \sigma_{y_i}^*)$ closest to (x_j, y_j)

    $r \leftarrow \max(\sigma_{x_i}^*, \sigma_{y_i}^*) \times 3$

    $x_1 \leftarrow \max(x_i^* - r, 0)$, $x_2 \leftarrow \min(x_i^* + r + 1, W)$

    $y_1 \leftarrow \max(y_i^* - r, 0)$, $y_2 \leftarrow \min(y_i^* + r + 1, H)$

    $X, Y \leftarrow \text{Meshgrid}([x_1, x_2), [y_1, y_2))$

    $G(x, y) \leftarrow \exp\left(-\frac{(x-x_j)^2}{2(\sigma_{x_i}^*)^2} - \frac{(y-y_j)^2}{2(\sigma_{y_i}^*)^2}\right)$

    $H_k \leftarrow H_k + G(X, Y)$

  end for

end for

return $\{H_k\}_{k=1}^{K}$

- 16, attention sequence videos created based on real radiologists eye gazing for different medical labels defined in CheXbert [43] are shown.

B.2 Foundation GazeX and Problem Formulation

We view an expert radiologist as a *stochastic policy* that generates a gaze scan-path G and the corresponding findings M, conditioned on a chest X-ray image I:

$$R : I \longmapsto p_R(G, M \mid I). \tag{S4}$$

The noise and long sequences in the expert behavior distribution can be reduced by processing the gaze scan-path to attention maps $\{\{H_k\}_{k=1}^{K_m}\}_{m \in M}$, and gaze-guided attention sequence video V:

$$p_R(\{\{H_k\}_{k=1}^{K_m}\}_{m \in M}, V, M \mid I). \tag{S5}$$

Let f_θ represent our proposed Gaze-X model, parameterized by θ. The goal is to mimic expert behavior by minimizing the divergence between the expert policy p_R and the model policy p_θ. However, accurately estimating the true gaze behavior of radiologists is difficult due to technical noise in data collection. Nevertheless, radiologists' gaze behavior is inherently structured: they are trained to inspect specific areas for particular findings, follow defined inspection sequences when examining X-ray images or anatomical regions, and apply global reasoning during the process. To reflect this, we decompose the expert policy into three complementary components: 1) Fine-grained Visual Perception (FVP): Precisely localizes attention for each medical finding by predicting spatial regions (bounding boxes and centroids) on the chest X-ray image. 2) Gaze Trajectory Mimicking (GTM): Replicates the radiologist's eye-tracking path to generate medical findings, capturing the temporal sequence of visual

attention across the X-ray image. 3) Sequential Dependency Awareness (SDA): Models the clinically relevant order of inspecting findings, predicting a permutation that reflects the radiologist's structured diagnostic reasoning process. Following this, the training process can be summarized as follows:

$$f_\theta^{\text{FVP}} : (I, M) \longrightarrow \{\{\hat{B}_k, \hat{c_k}\}_{k=1}^{K_m}\}_{m\in M} \cup \{\hat{B}_m, \hat{c_m}\}_{m\in M}, \tag{S6}$$

$$f_\theta^{\text{GTM}} : (I, V, V_{disease}) \longrightarrow \hat{M}, \tag{S7}$$

$$f_\theta^{\text{SDA}} : (I, \tilde{V}) \longrightarrow \hat{\pi}, \tag{S8}$$

where for each medical finding $m \in M$, let $\hat{B}_m$ and $\hat{c}_m$ denote the predicted bounding box and centroid, respectively, for the entire observation area. For visual clusters associated with finding m, let $\{\hat{B}_k, \hat{c}_k\}_{k=1}^{K_m}$ represent the corresponding predicted bounding boxes and centroids, where K_m is the number of clusters specific to m. Additionally, $\hat{M}$ is the set of predicted medical findings, $V_{\text{disease}} \subseteq V$ is the subset of frames extracted from V that are associated with a specific abnormality description, and $\hat{\pi}$ is the predicted permutation order.

Each part is trained with its dedicated objective detailed in Sections B.2.1, B.2.2, and B.2.3, respectively.

B.2.1 Fine-Grained Visual Perception

The fine-grained visual perception (FVP) component enables GazeX to localize radiologist fixations by predicting the bounding boxes and centroids for each medical finding (Eq. (S6)). This module learns the spatial correspondence between textual medical findings and their anatomical locations, which enables spatial grounding of findings, allowing the model to interpret medical concepts in relation to their anatomical context. Given a chest X-ray image I and a set of medical findings $m \in M$, the model is trained to perform the following steps in a structured format:

1. **Visual Cluster Prediction**: For each medical finding m, the model outputs a set of visual clusters characterized by their predicted bounding boxes and attention centroids, denoted as $\{\hat{B}_k, \hat{c}_k\}_{k=1}^{K_m}$, where K_m is the number of clusters specific to m.

2. **Observation Area Prediction**: Using the visual clues from the predicted clusters, the model generates the final observation area for each m, represented by the predicted bounding box $\hat{B}_m$ and attention centroid $\hat{c}_m$ in structured format: ⟨answer⟩$(x_1, y_1), (x_2, y_2)$,(centroid$_x$, centroid$_y$)⟨/answer⟩.

The model learns this spatial grounding through a cross-entropy loss over the structured output sequence:

$$\mathcal{L}_{FVP} = -\sum_{m\in M}^{|M|} \log p_\theta(\{(B_k, c_k)\}_{k=1}^{K_m}, (B_m, c_m)|I, m) \tag{S9}$$

where p_θ denotes the model's conditional probability of generating the correct spatial localization given the image I and finding m.

This supervision approach allows the model to learn precise mappings between medical findings and their corresponding anatomical regions, ensuring that the predicted localizations align with expert radiologist attention patterns. The structured output format facilitates easy extraction of spatial coordinates while maintaining the model's ability to ground textual findings in visual anatomy.

B.2.2 Gaze Trajectory Mimicking

While the FVP module equips Gaze-X with the ability to spatially ground medical findings, it does not capture the comprehensive and structured examination process followed by expert radiologists. To address this, we introduce the gaze trajectory mimicking (GTM) component, which enables Gaze-X to generate findings by emulating the temporal progression of expert visual attention on a X-ray image, as captured in the gaze-guided attention video (Eq. (S7)).

Given a chest X-ray image I and the corresponding attention sequence video V, the model is trained to produce the set of medical findings $\hat{M}$ in a way that respects the implicit visual reasoning path encoded in V. This process is supervised using the ground truth findings M, and optimized via the following objective:

$$\mathcal{L}_{GTM} = -\sum_i \log p_\theta(M_i \mid I_i, V_i), \tag{S10}$$

where i indexes individual training examples, and p_θ denotes the distribution of the model over medical findings conditioned on the attention sequence video. In addition to training the model with individual diagnosis attention sequence videos, we incorporate disease-specific attention sequence videos $V_{disease}$. This approach enhances the model's reasoning for disease-based or anatomical region-based visual analysis and minimizes the influence of mimicking individual radiologist inspection patterns.

By aligning the prediction process with the temporal structure of expert gaze behavior, GTM encourages the model to not only identify relevant findings but also to infer them in a clinically meaningful order, thereby enhancing the interpretability of the output.

B.2.3 Sequential Dependency Awareness

While the FVP and GTM modules equip Gaze-X with spatial grounding and comprehensive visual reasoning, they do not account for the clinical dependencies between the regions. Radiologists often follow an interpretable order when scanning pathologically related areas. To model this behavior, we introduce the sequential dependency awareness (SDA) module, which enables Gaze-X to recover the expert's intended viewing sequence (Eq. (S8)).

Given a chest X-ray image I and a permuted version of the attention sequence video $\widetilde{V}$, the model is trained to predict the permutation order $\hat{\pi}$ that align with the original expert viewing sequence. Supervision is provided using ground truth permutation π, and training is carried out to minimize the following objective:

$$\mathcal{L}_{SDA} = -\sum_{i} \log p_\theta(\pi_i \mid I_i, \tilde{V}_i), \tag{S11}$$

where i represents each training sample and p_θ represents the model-induced distribution over plausible viewing sequences conditisoned on the image and the permuted video. Similar to GTM, we incorporate disease-specific attention sequence videos into SDA training.

By learning to recover the clinically meaningful order of attention, the SDA module fosters consistency in the model output, mirroring the structured diagnostic process of expert radiologists.

B.2.4 Objective Combination and Update

With each component capturing a distinct aspect of expert behavior: spatial grounding (FVP), comprehensive gaze-driven reasoning (GTM), and temporal structure (SDA). We jointly train GazeX using a weighted combination of the three corresponding loss terms.

$$\mathcal{L}(\theta) = \lambda_1 L_{FVP} + \lambda_2 L_{GTM} + \lambda_3 L_{SDA} \tag{S12}$$

Thus, the Gaze-X parameters θ can be optimized as θ' using:

$$\theta' = \arg\min_{\theta} \big(\lambda_1 L_{FVP} + \lambda_2 L_{GTM} + \lambda_3 L_{SDA}\big), \tag{S13}$$

with hyperparameters λ_i are weighted coefficients for each component.

The Foundation Gaze-X framework is designed to mimic expert radiologist behavior by jointly modeling spatial attention, diagnostic trajectory, and scan path structure. This is achieved through a modular architecture that incorporates three specialized components: 1) FVP aligns visual features with textual findings through gaze-supervised spatial localization. 2) GTM leverages temporal attention sequence video to guide the generation of structured medical findings. 3) SDA learns to reconstruct the clinically meaningful order in which a radiologist inspects the X-ray image.

By integrating these complementary objectives, Gaze-X not only predicts accurate diagnostic output, but also exhibits human-aligned interpretable behavior. This structured training strategy reflects the inherent complexity nature of expert visual reasoning in medical imaging, providing a foundation for more explainable diagnostic systems.

Moreover, aligning the model with expert gaze behavior paves the way for clinical applications where trust and interpretability are essential, such as visual grounding, visual question answering, and report generation.By modeling both what radiologists observe and how they examine medical images, GazeX produces outputs that are not only diagnostically accurate but also contextually and visually grounded. This design enhances transparency and fosters user confidence in downstream decision-making tasks, making GazeX a more reliable assistant in real-world clinical workflows.

B.3 Implementation Details

B.3.1 Rationale for Selecting Qwen2-VL as the Backbone

Open-source accessibility and multimodal fusion. Qwen2-VL is a fully open-source vision–language model that permits parameter-level fine-tuning on our heterogeneous multimodal medical data, including chest X-ray images, radiologist gaze-guided attention videos, and radiologist-generated text reports, while natively supporting multimodal fusion within a unified architecture. This openness is essential for reproducibility and domain adaptation in medical AI.

Unified positional encoding via M-RoPE. Qwen2-VL introduces Multimodal Rotary Position Embedding (M-RoPE), which overcomes the limitation of conventional RoPE in encoding only 1D sequential positions. By decomposing the rotary embedding into three components corresponding to the temporal, height, and width axes, M-RoPE enables the language model to simultaneously capture and integrate the 1D positional structure of text, the 2D spatial structure of X-ray images, and the 3D spatiotemporal structure of gaze videos within a unified positional framework [44]. This property is particularly well-aligned with our task, as radiologist gaze trajectories are inherently spatiotemporal, encoding both *where* and *when* the radiologist looked, and must be jointly grounded with 2D image content and 1D textual findings.

B.3.2 Qwen2-VL pre-training

We train the Qwen2-VL models with 2 billion parameters on 1 compute node of 2 NVIDIA A100 GPUs. We use a training batch size of 4 (2 per GPU), the AdamW optimizer, base learning rate 1×10^{-5} and a cosine learning-rate scheduler with a linear warm-up of 0.1 ratio. We down-sample the original image and each video frame by a factor of 4 in both height and width, and set the frame rate to 1 FPS for model training. For training and evaluation, we adopt an 80/20 split over the full set of 3,032 eye gazing recordings. After the data-processing pipeline, more than 13,000 expert videos are extracted and used.

B.3.3 Downstream evaluation tasks

The implementation details for the training and evaluation of each downstream network presented are provided below:

Radiology report generation We evaluate the report generation task on 1 compute node of 2 NVIDIA H100 GPU. We use a training batch size of 16 (8 per GPU), AdamW optimizer, base learning rate 1×10^{-4}, and a cosine learning rate scheduler. Low-Rank Adaptation [45] (LoRA) for large language models is used for the downstream task fine-tuning with LoRA rank 16, targeting only the language model.

Visual-question answering We evaluate the visual-question answering task on 1 compute node of 2 NVIDIA H100 GPU. We use a training batch size of 16 (8 per GPU), AdamW optimizer, base learning rate 1×10^{-4}, and a cosine learning rate scheduler. Low-Rank Adaptation [45] (LoRA) for large language models is used for the downstream task fine-tuning with LoRA rank 8, targeting only the language model.

Visual Grounding We evaluate the visual grounding task on 1 compute node of 2 NVIDIA H100 GPU. We use a training batch size of 16 (8 per GPU), AdamW optimizer, base learning rate 1×10^{-4}, and a cosine learning rate scheduler. Low-Rank Adaptation [45] (LoRA) for large language models is used for the downstream task fine-tuning with LoRA rank 8, targeting only the language model.

B.3.4 Baseline models for downstream tasks performance comparison

The source code and pretrained weights for the baseline models in the report generation task were obtained from public resources, including R2Gen [46], CMN [47], RGRG [48], R2GenGPT [49], and PromptMRG [50]. In contrast, neither official code nor pretrained weights are available for MA [51] or STREAM [52]. Traditional encoder-decoder models, such as R2Gen [46], generate free-form reports directly from input images without explicitly modeling spatial information. Although effective for general captioning, this approach overlooks correspondences between findings and anatomical regions, thereby reducing sensitivity to fine-grained image details.

Region-enhanced encoder-decoder models, MA [51], CMN [47], and PromptMRG [50], explicitly integrate regional features (e.g., lesion patches or organ-level segments) into the encoder. Nevertheless, their reasoning remains opaque: while they can associate visual patches with disease presence, they fail to explain why diseases manifest in specific locations or why certain regions are diagnostically critical. Prompt-driven large language models, including STREAM [52], R2GenGPT [49], and RGRG [48], leverage LLMs for report generation. However, spatial information is encoded in a limited manner, resulting in loose vision-language alignment. These models employ diverse architectures for visual feature extraction and decoding. ResNet serves as the visual encoder in R2Gen, CMN, and PromptMRG, while DenseNet is used by MA to capture dense representations. RGRG adopts Faster R-CNN for region-specific features via bounding-box detection. Vision Transformers are employed by R2GenGPT and STREAM to model global image context through self-attention. For decoding, RGRG, R2GenGPT, and STREAM integrate LLMs to produce coherent reports, exploiting their advanced generative capabilities. For visual-question answering and visual grounding tasks, all foundational and deep learning models were reimplemented from their official released codebases. For the visual question answering task, baseline models include the specialized Medical-CXR-VQA [3] and several multi-modal medical large language foundation models: LLaVA-Med-7B [53], RadFM-14B [23], HuatuoGPT-Vision-7B [24], HuatuoGPT-Vision-32B [24], Bio-Medical-MultiModal-Llama-8B [54], MedGemma-4B-it [55], Lingshu-7B [56], and Lingshu-32B [56]. RadFM is primarily pretrained on radiologic modalities and anatomical regions of the human body, whereas the others are trained on diverse medical data, including non-radiologic modalities such as microscopy images, ultrasound and dermatology images. For visual grounding task, we selected models with different training methods, including ChEX [57] (visual grounding foundation model), TransVG [58] (supervised learning model for visual grounding), BioVIL [4] (CLIP-based learning model for visual grounding), and OmniFM-DR [59] (generative visual-grounding model). For each baseline, we used its corresponding image preprocessing pipeline and image inference implementation, if provided. We initially used exact matching for the visual question-answering task. However, since the foundation model often failed to produce the exact answer required even we manually designed the prompt, we adopted decomposition and applied correction unification before matching.

B.3.5 Comparison to other gaze-based models

Recent work has produced a growing body of gaze-assisted chest X-ray interpretation models [60; 61; 62], most of which focus on analyzing patterns in radiologist gaze and leveraging them for downstream tasks. For instance, FixationFormer [61] uses radiologist gaze trajectories as an additional input modality to support image-based disease classification, but does not generate findings or any form of gaze-based explanation at inference time. LogitGaze-Med [62] proposes a framework for scanpath prediction; its central goal is to model and evaluate radiologist gaze trajectories under a target-present search condition. Accordingly, it predicts where radiologists would look given a specified diagnostic target, but does not produce diagnostic findings itself. Thinking with Gaze [60] incorporates gaze as supervision for visual reasoning, yet its model is trained with a classification head for disease prediction and does not generate free-text findings, thereby precluding report-level diagnosis. In contrast, GazeX is explicitly designed for explainable diagnosis generation. At inference time, it jointly predicts clinical findings and a temporally ordered sequence of attention heatmaps that together form a Gaze-Guided Attention Video, providing an interpretable visual rationale for each finding. Compared to existing gaze-based models, the GazeX framework is uniquely designed to (i) represent radiologist gaze trajectories as Gaze-Guided Attention Videos and (ii) train a vision–language model to jointly learn the association between these gaze videos and clinical findings, enabling the simultaneous generation of findings and their corresponding gaze-attention rationales at inference.

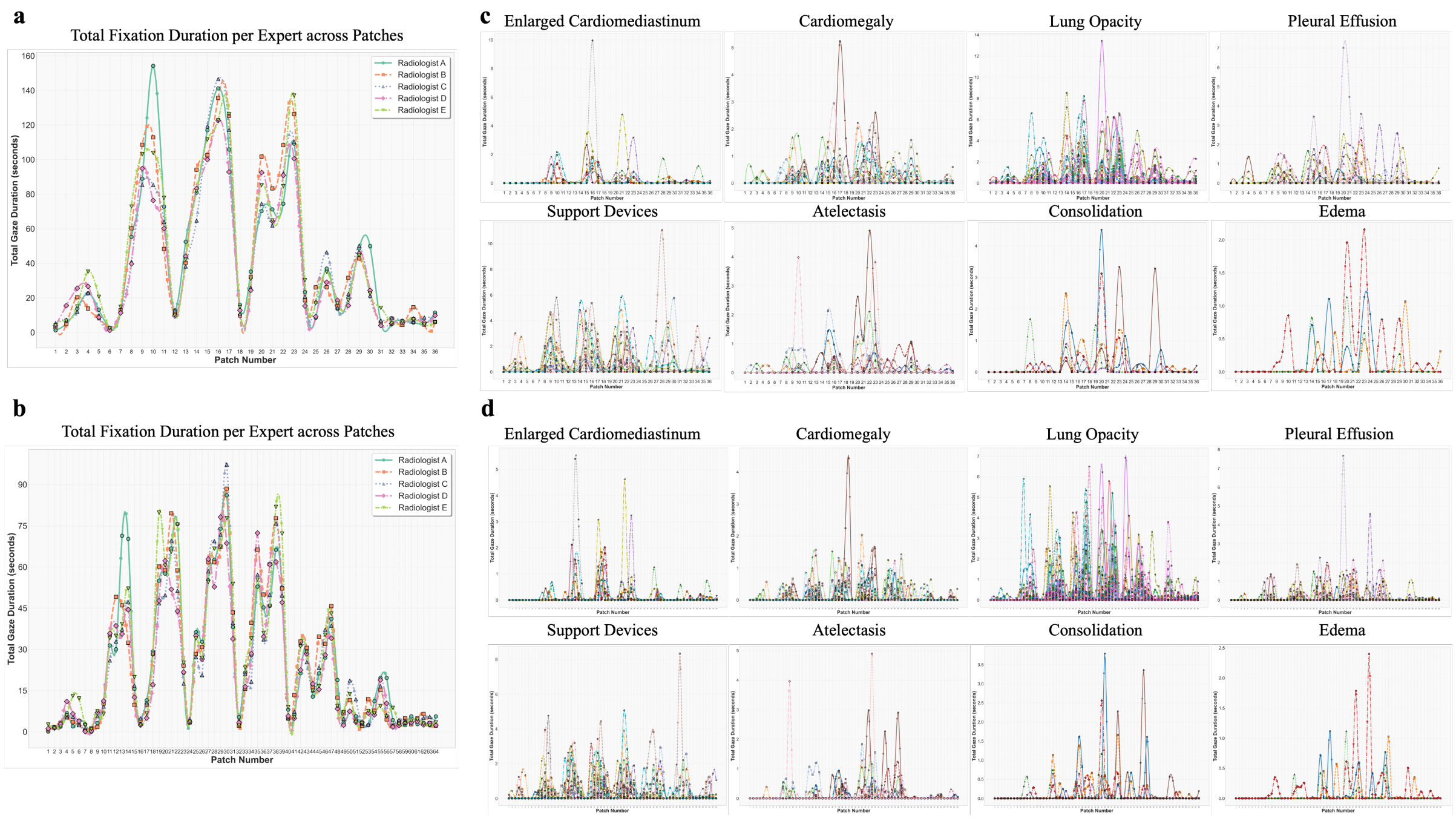

Supplementary Figure 1: **Total fixation duration across patches in different grid settings. a-b**, Total time spent across 6×6 and 8×8 grid of patches respectively, illustrating consistent fixation patterns and dwell times, with the central thoracic regions receiving the greatest attention. **d-c**, Total time spent per case across 6×6 and 8×8 grids of patches, stratified by representative case distributions across clinical categories.

a

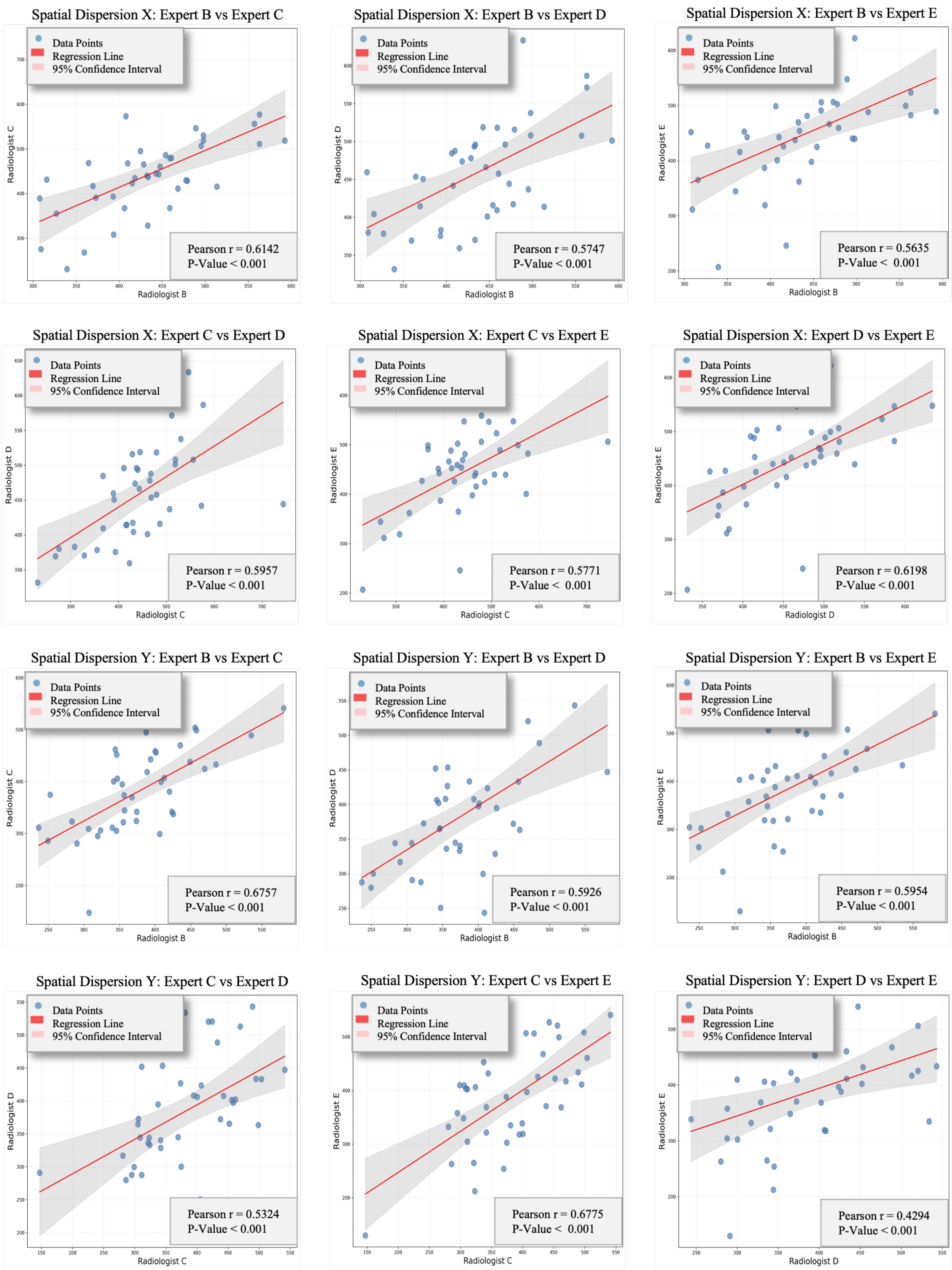

Supplementary Figure 2: **Scatter plots showing case-level dispersion of radiologists' fixation coordinates. a**, Case-level dispersion of fixation coordinates along both axes, revealing comparable spatial variability when attention is directed to similar anatomical regions.

a

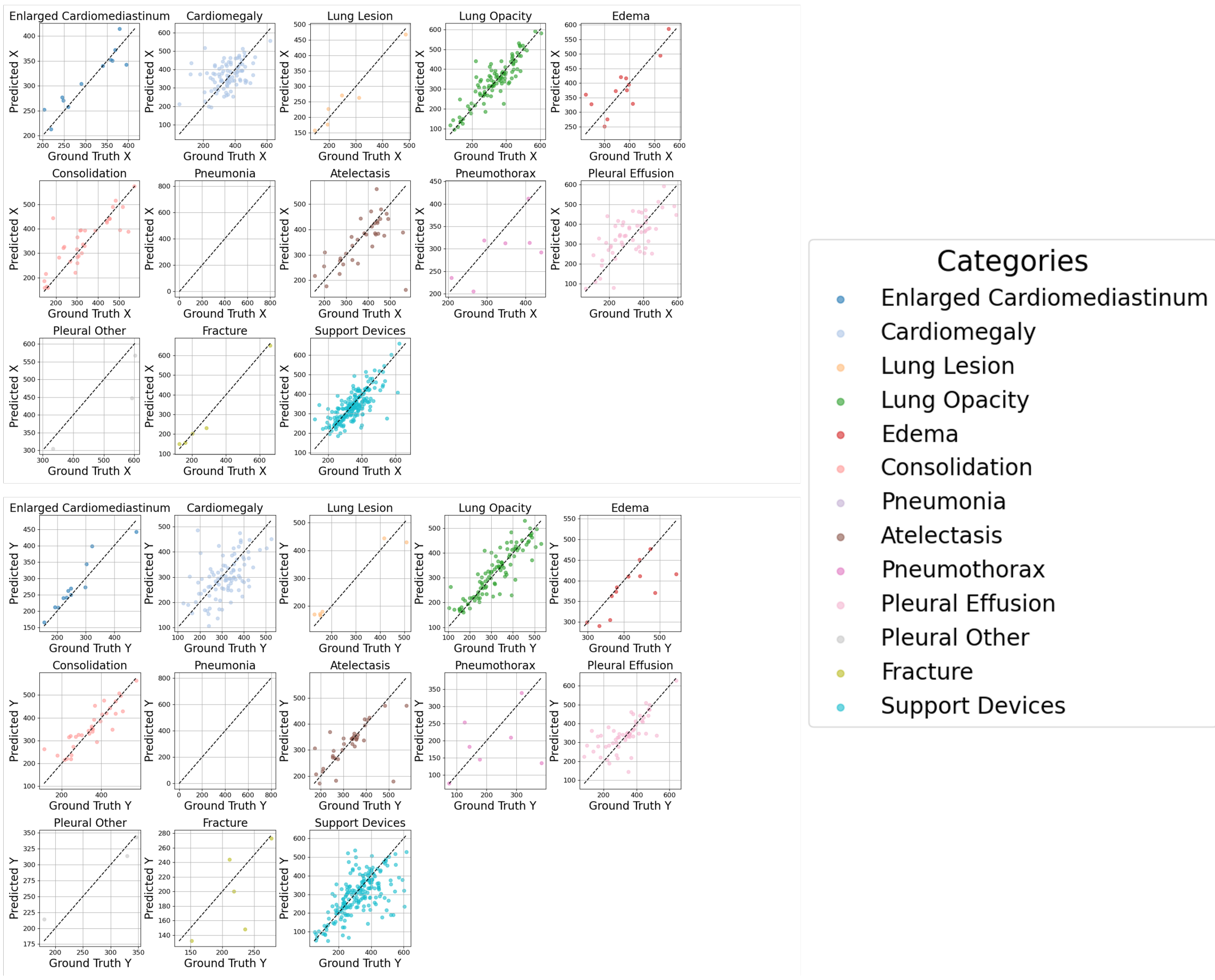

Supplementary Figure 3: **Scatter plots showing the agreement between GazeX predicted gaze centroids based on ground truth findings and those from radiologists. a**, Scatter plots of X and Y coordinates showing agreement between GazeX predicted gaze centroids based on ground truth findings and those from radiologists across 13 medical labels defined in CheXbert.

a

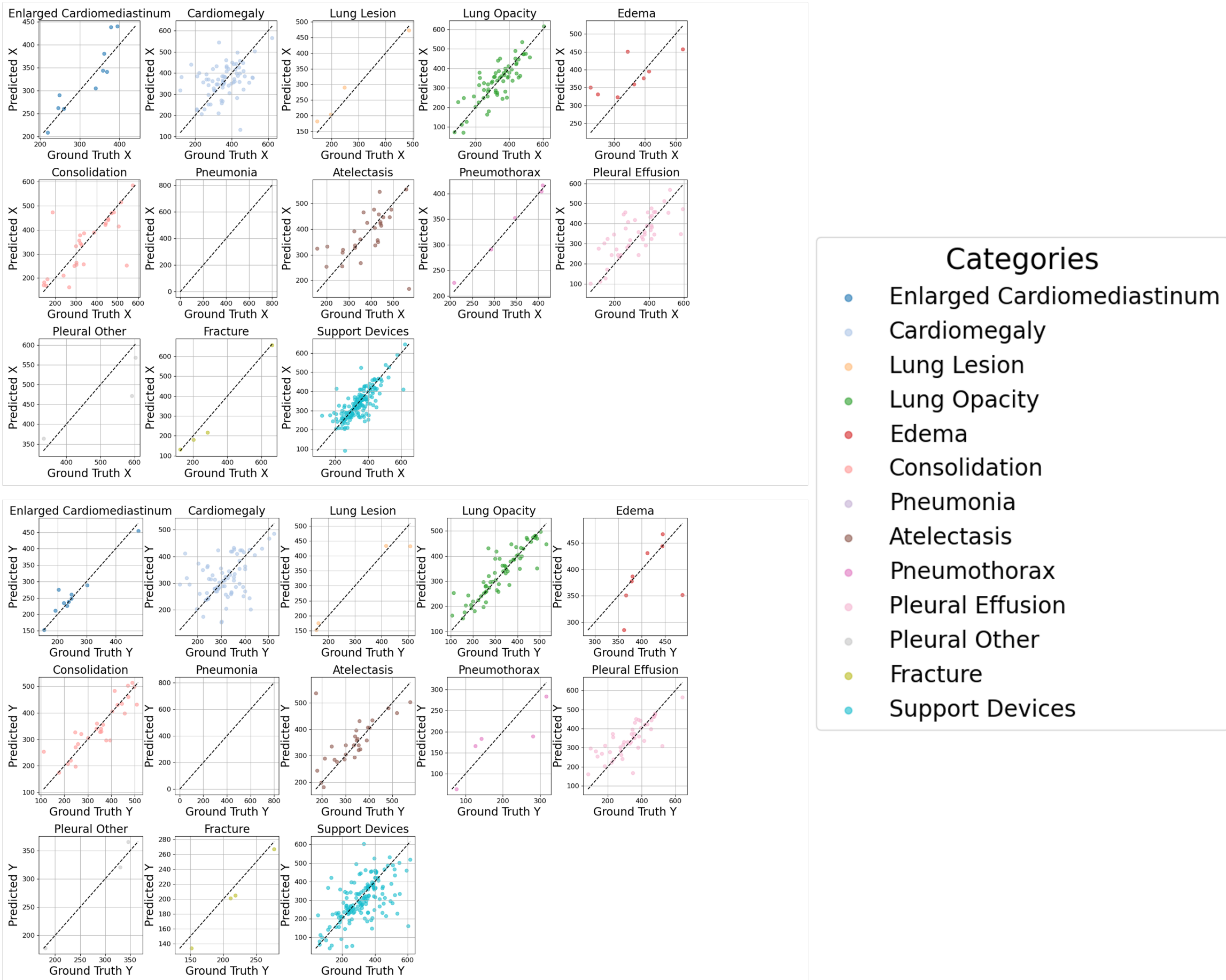

Supplementary Figure 4: **Scatter plots showing the agreement between GazeX predicted gaze centroids and those from radiologists.** **a**, Scatter plots of X and Y coordinates showing agreement between GazeX predicted gaze centroids based on GazeX generated findings and those from radiologists across 13 medical labels defined in CheXbert.

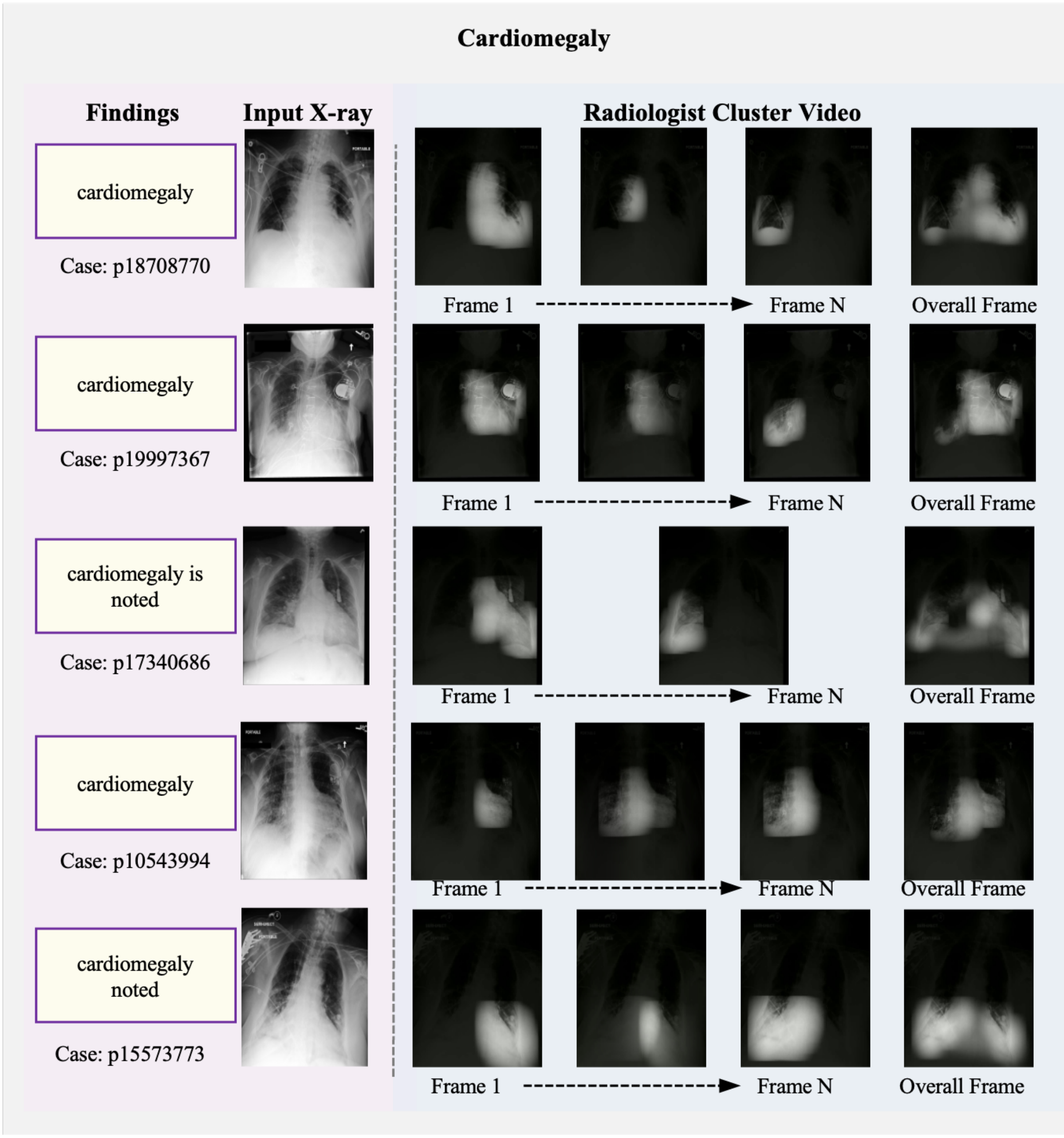

Supplementary Figure 5: **Gaze-guided attention videos of different training samples for cardiomegaly.**

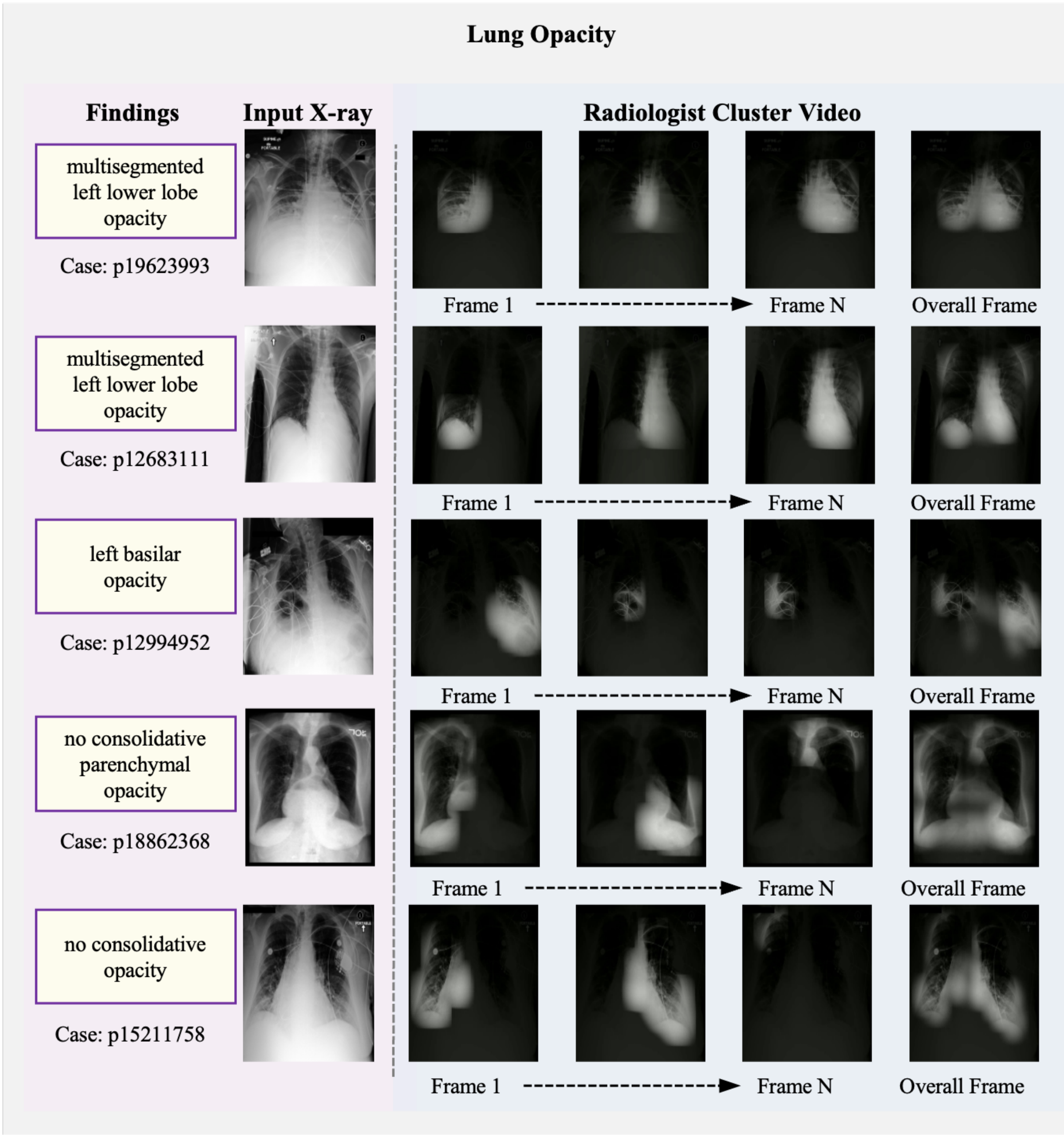

Supplementary Figure 6: **Gaze-guided attention videos of different training samples for lung opacity.**

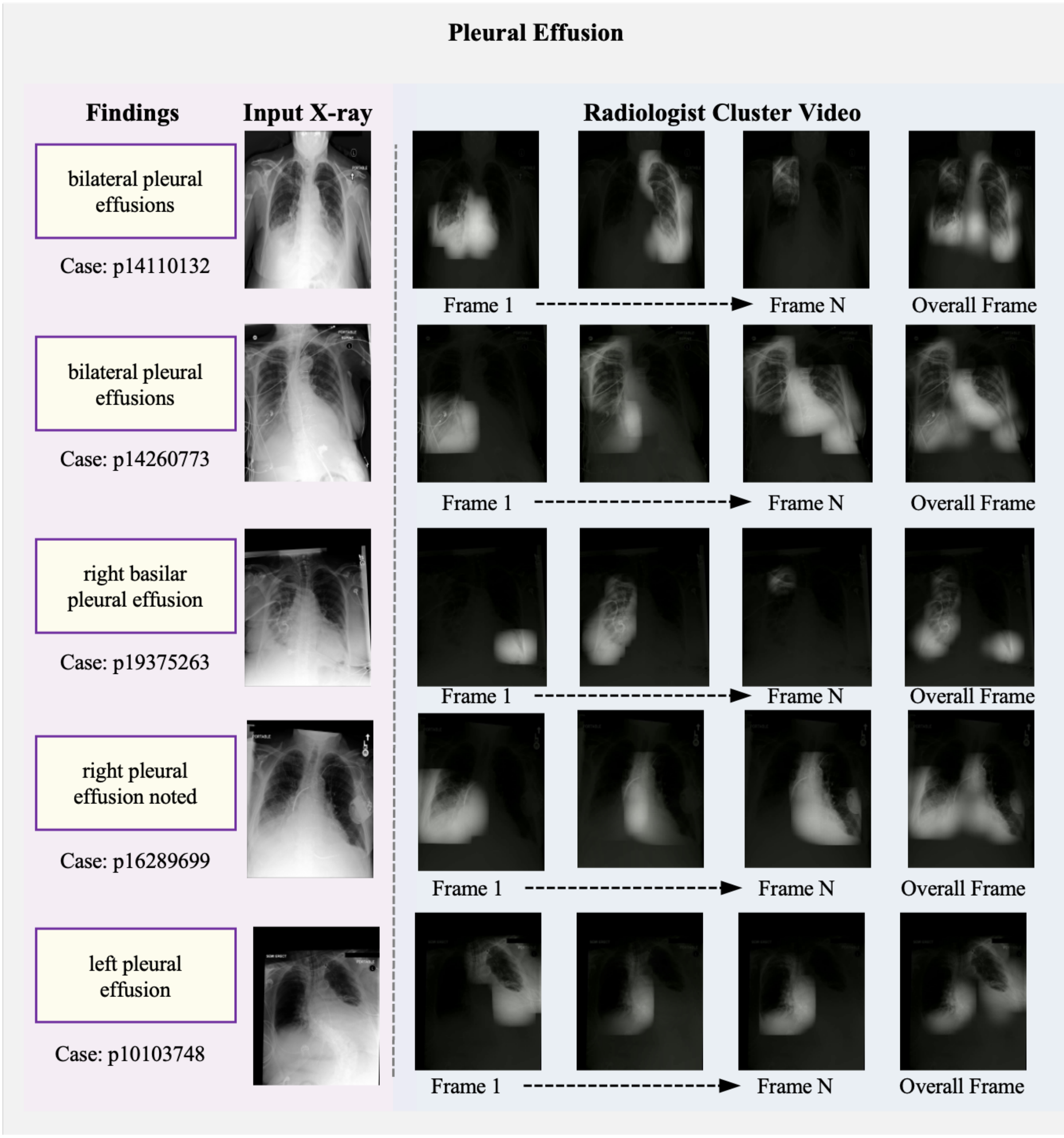

Supplementary Figure 7: **Gaze-guided attention videos of different training samples for pleural effusion.**

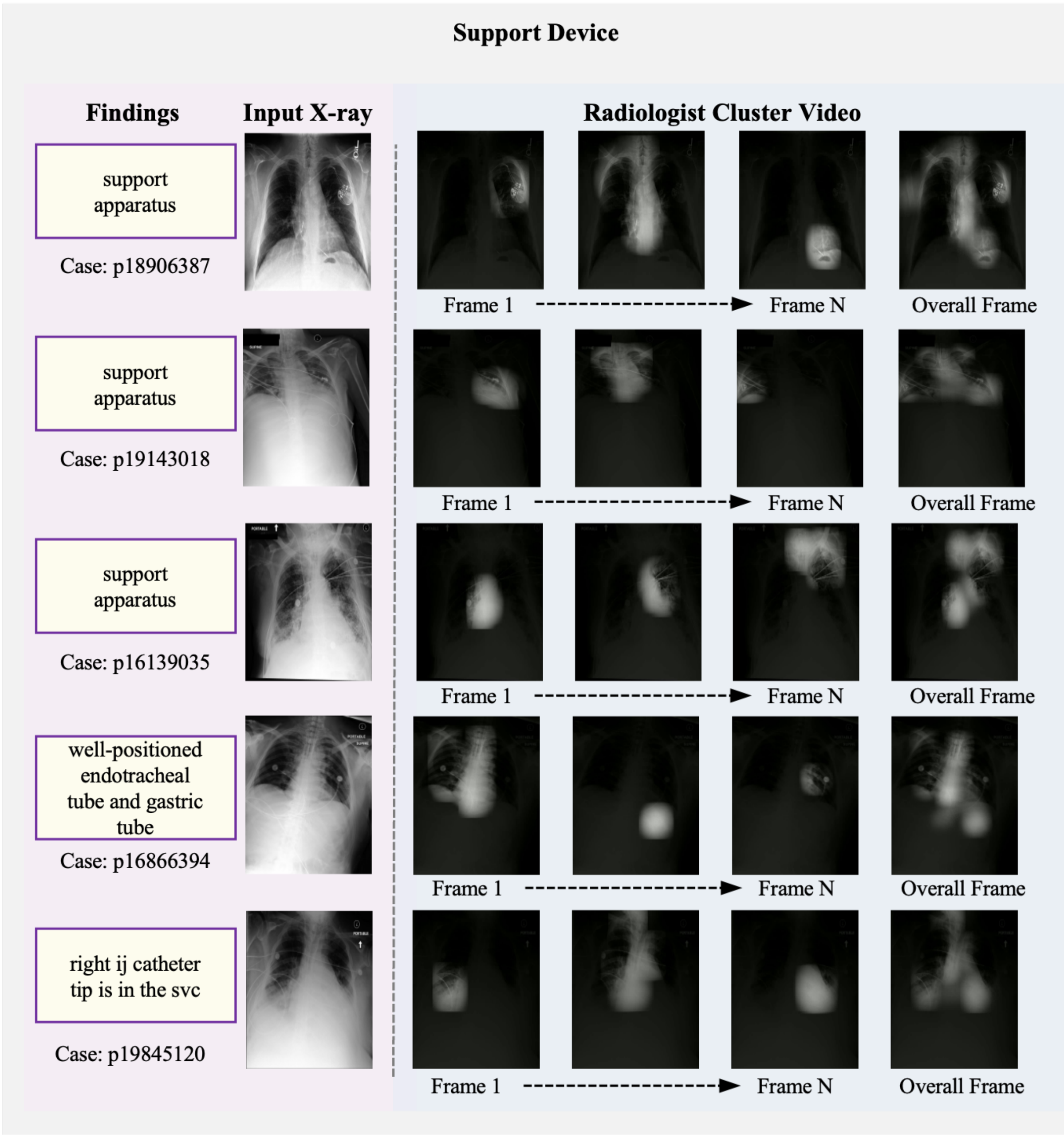

Supplementary Figure 8: **Gaze-guided attention videos of different training samples for support device.**

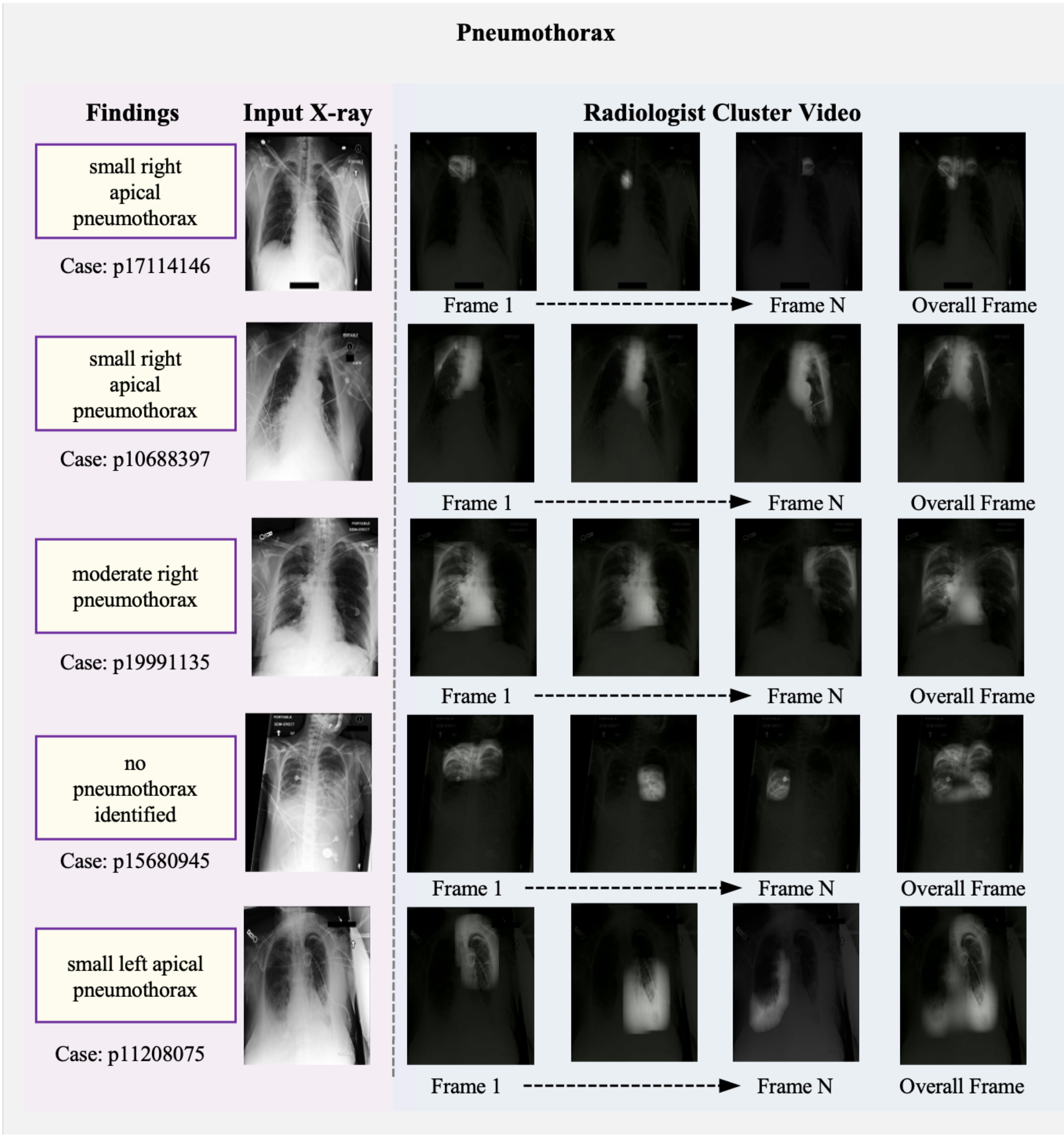

Supplementary Figure 9: **Gaze-guided attention videos of different training samples for pneumothorax.**

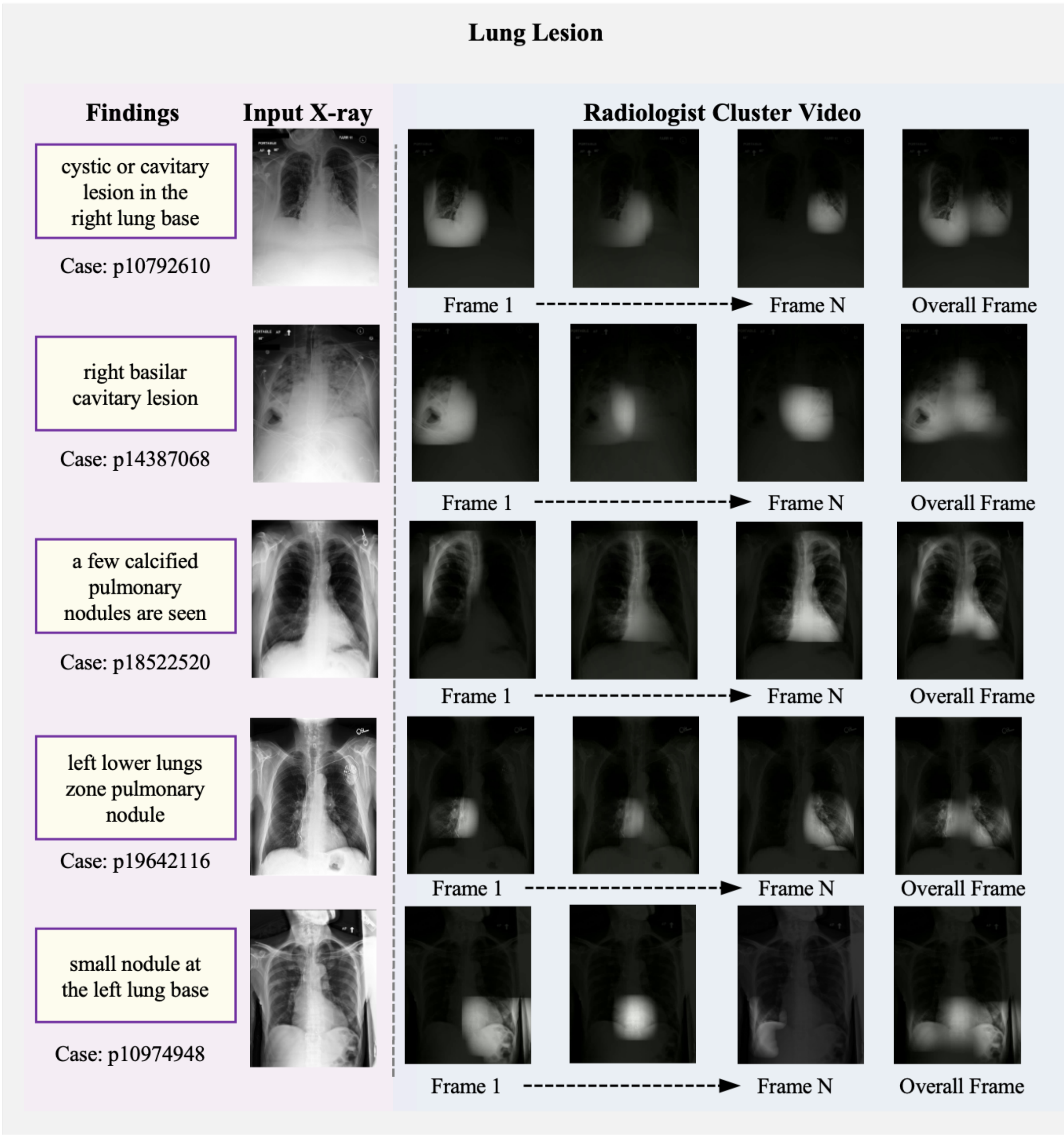

Supplementary Figure 10: **Gaze-guided attention videos of different training samples for lung lesion.**

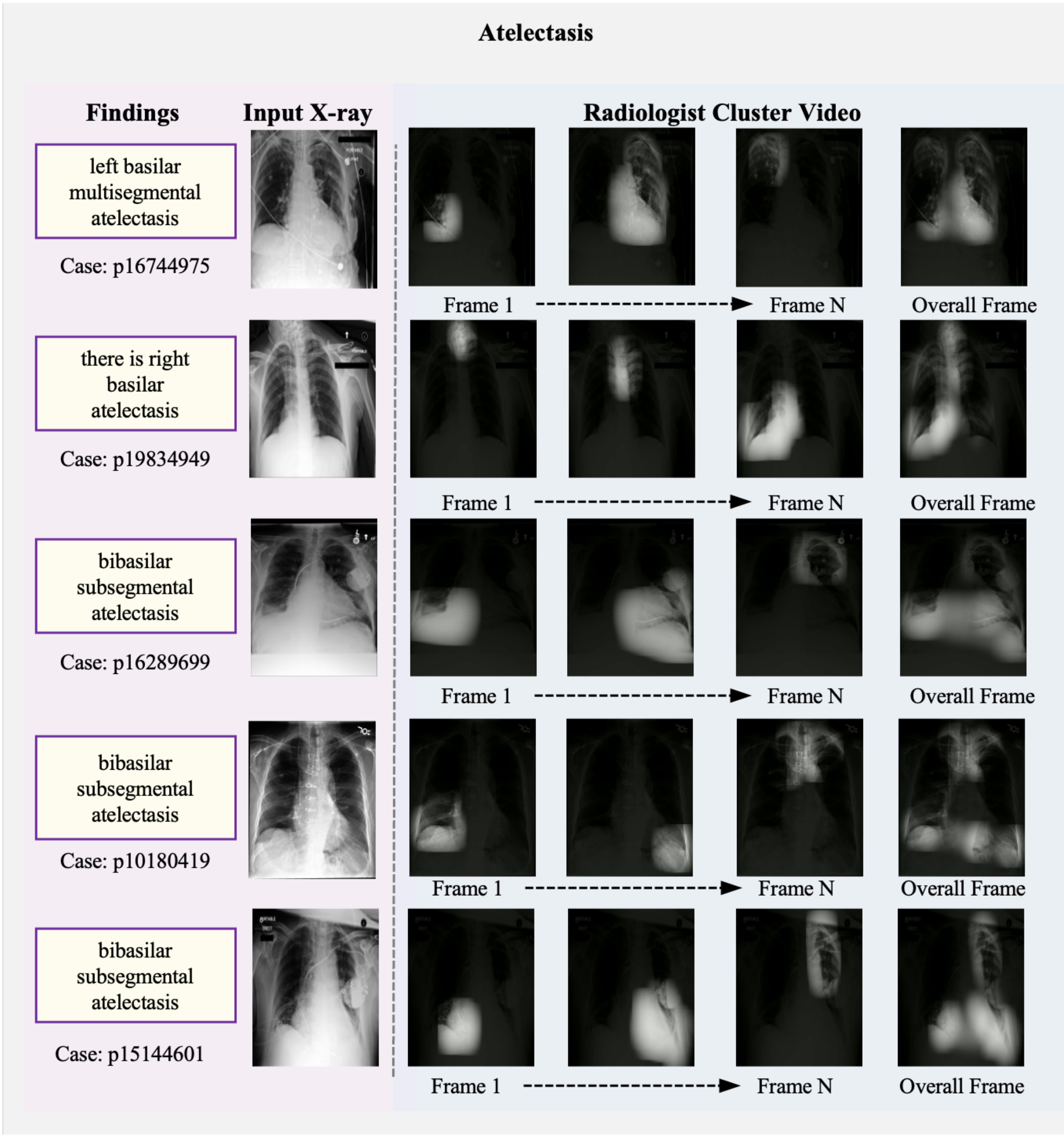

Supplementary Figure 11: **Gaze-guided attention videos of different training samples for atelectasis.**

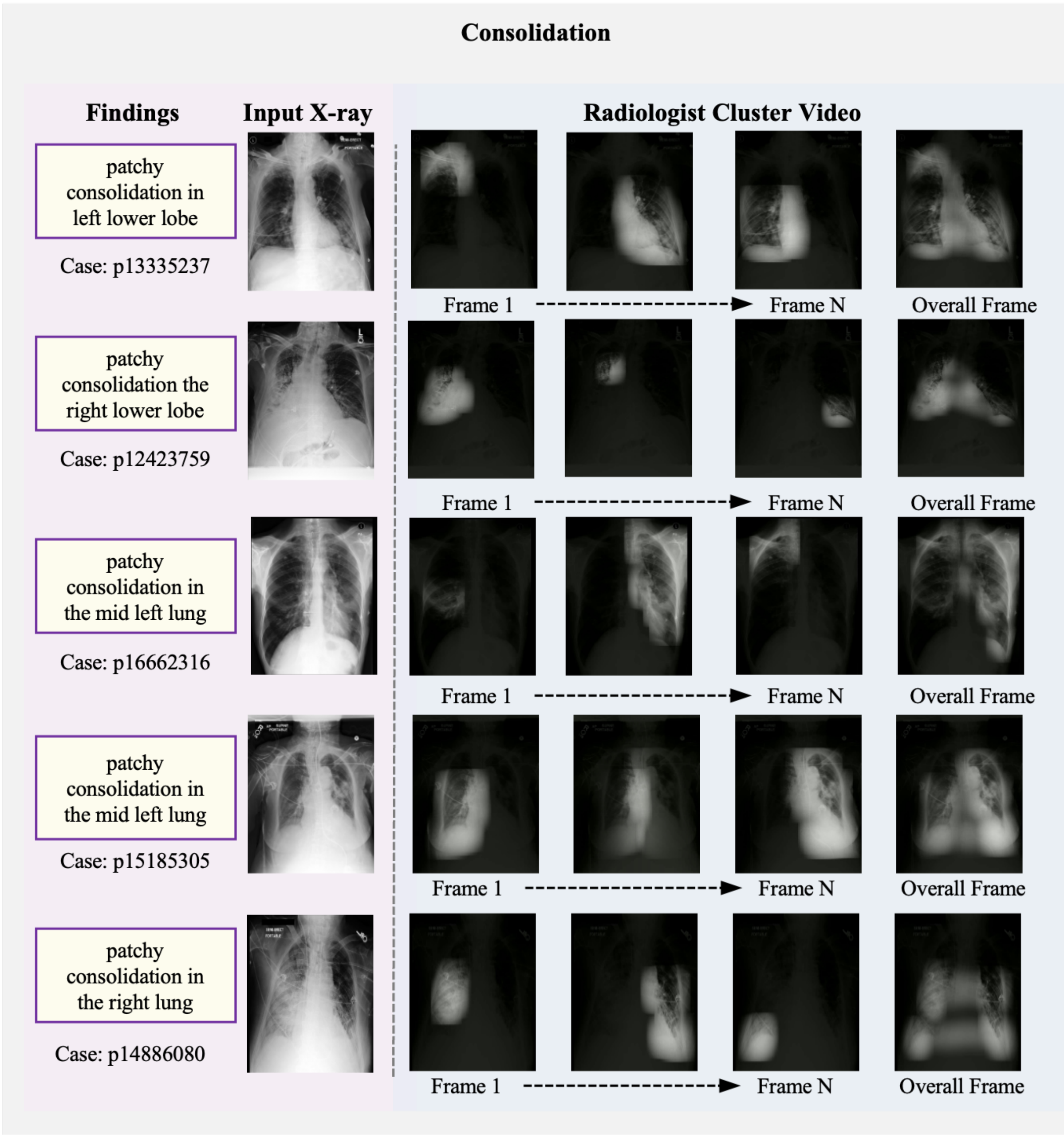

Supplementary Figure 12: **Gaze-guided attention videos of different training samples for consolidation.**

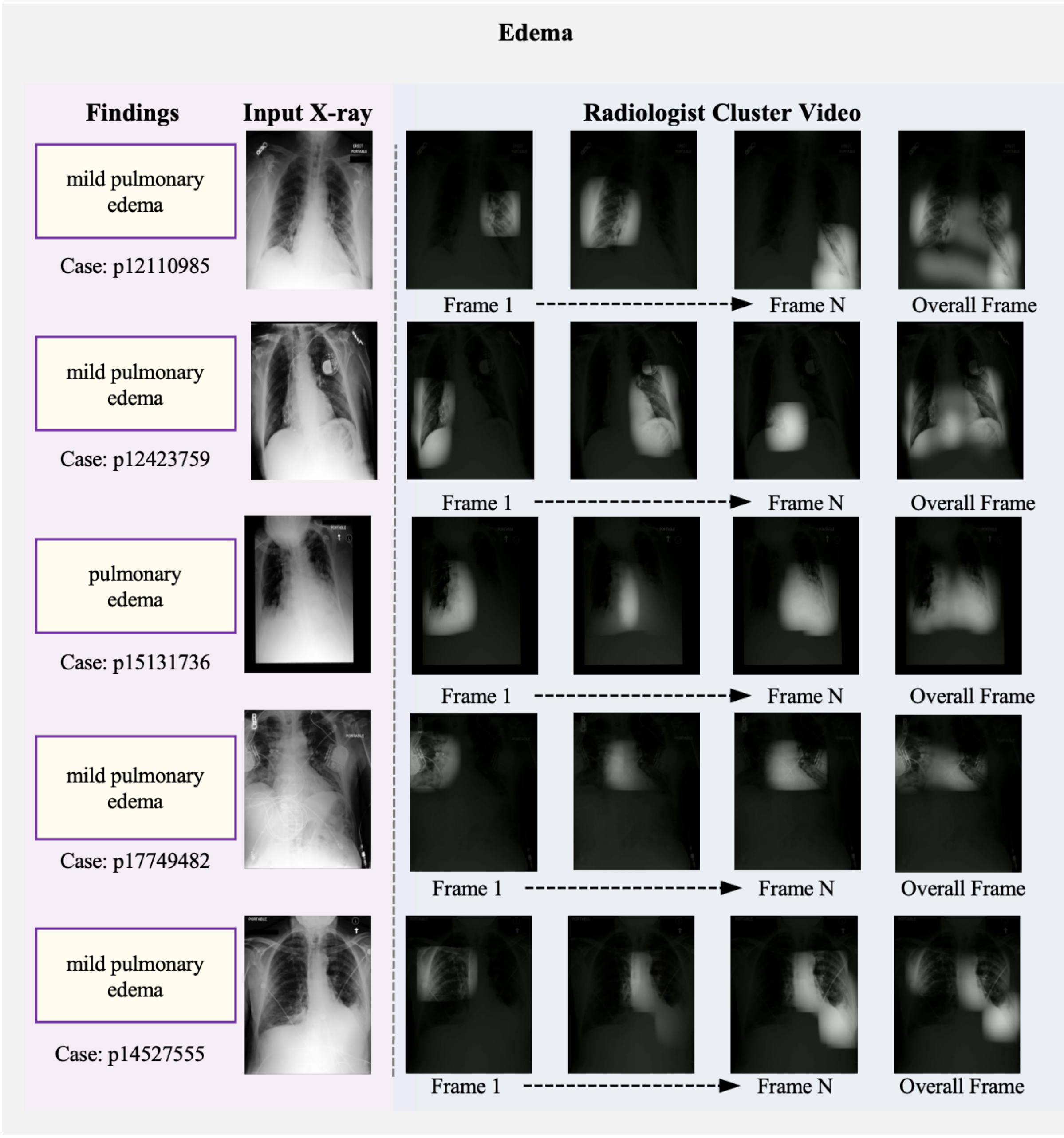

Supplementary Figure 13: **Gaze-guided attention videos of different training samples for edema.**

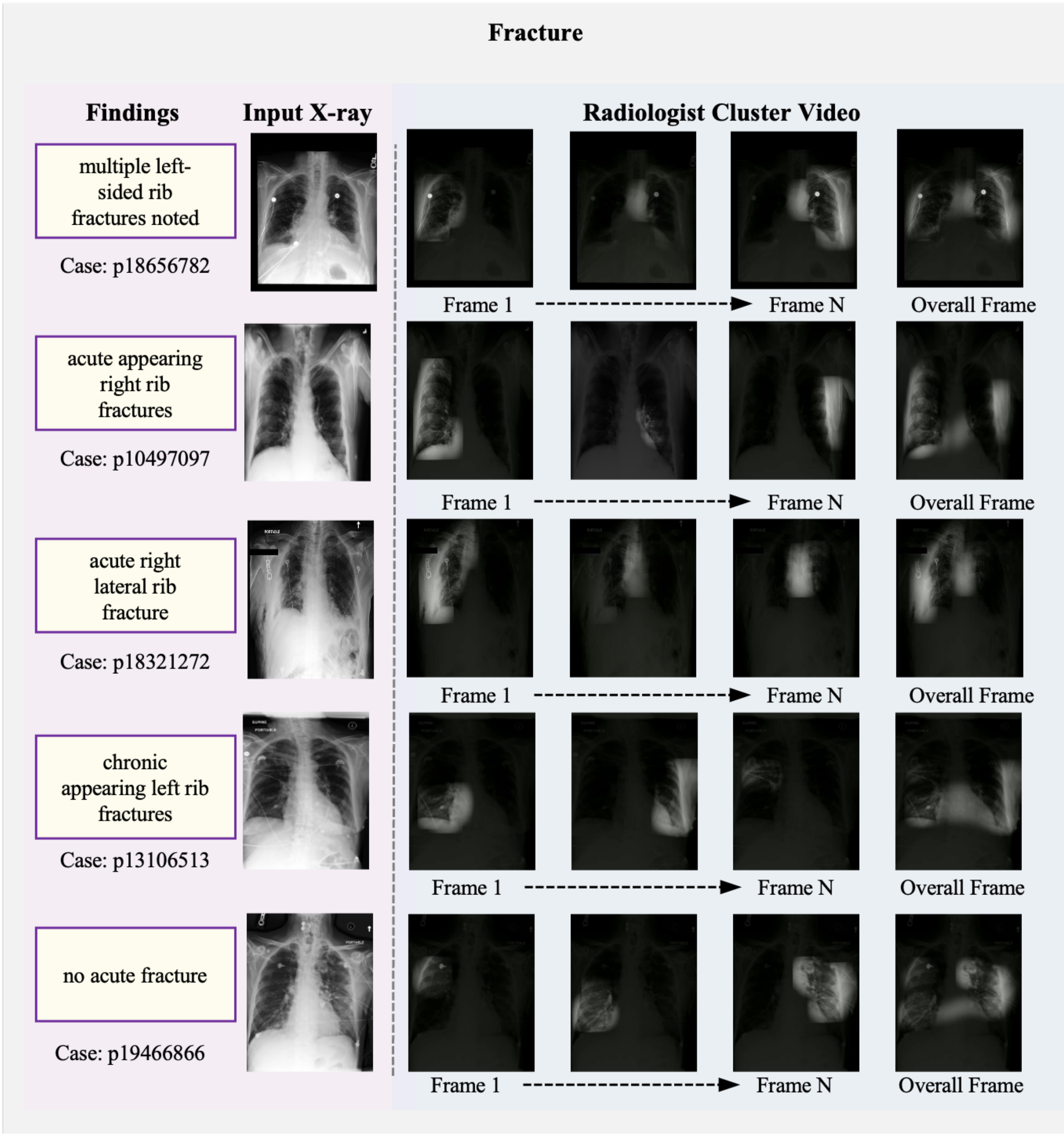

Supplementary Figure 14: **Gaze-guided attention videos of different training samples for fracture.**

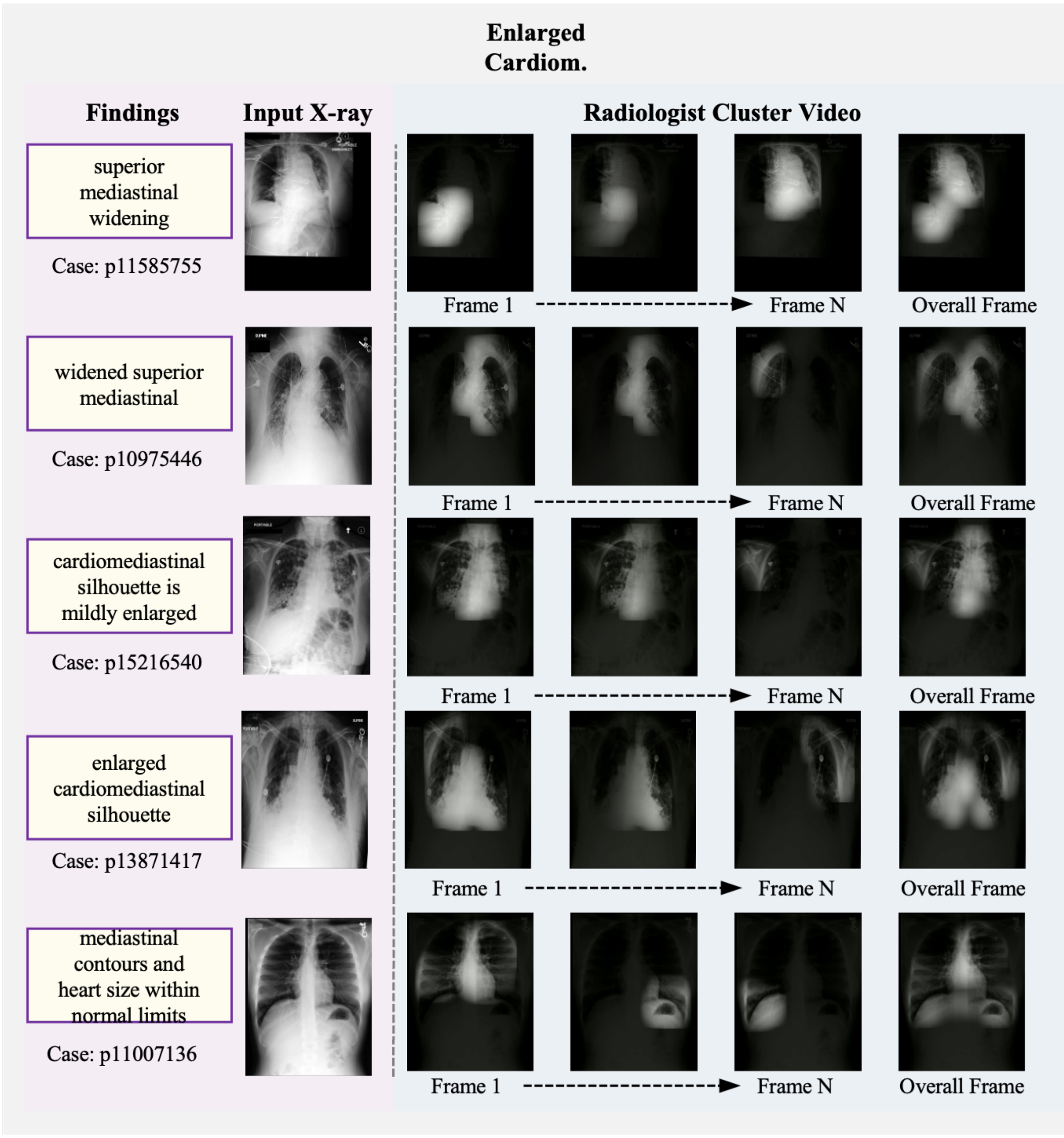

Supplementary Figure 15: **Gaze-guided attention videos of different training samples for enlarged cardiom..**

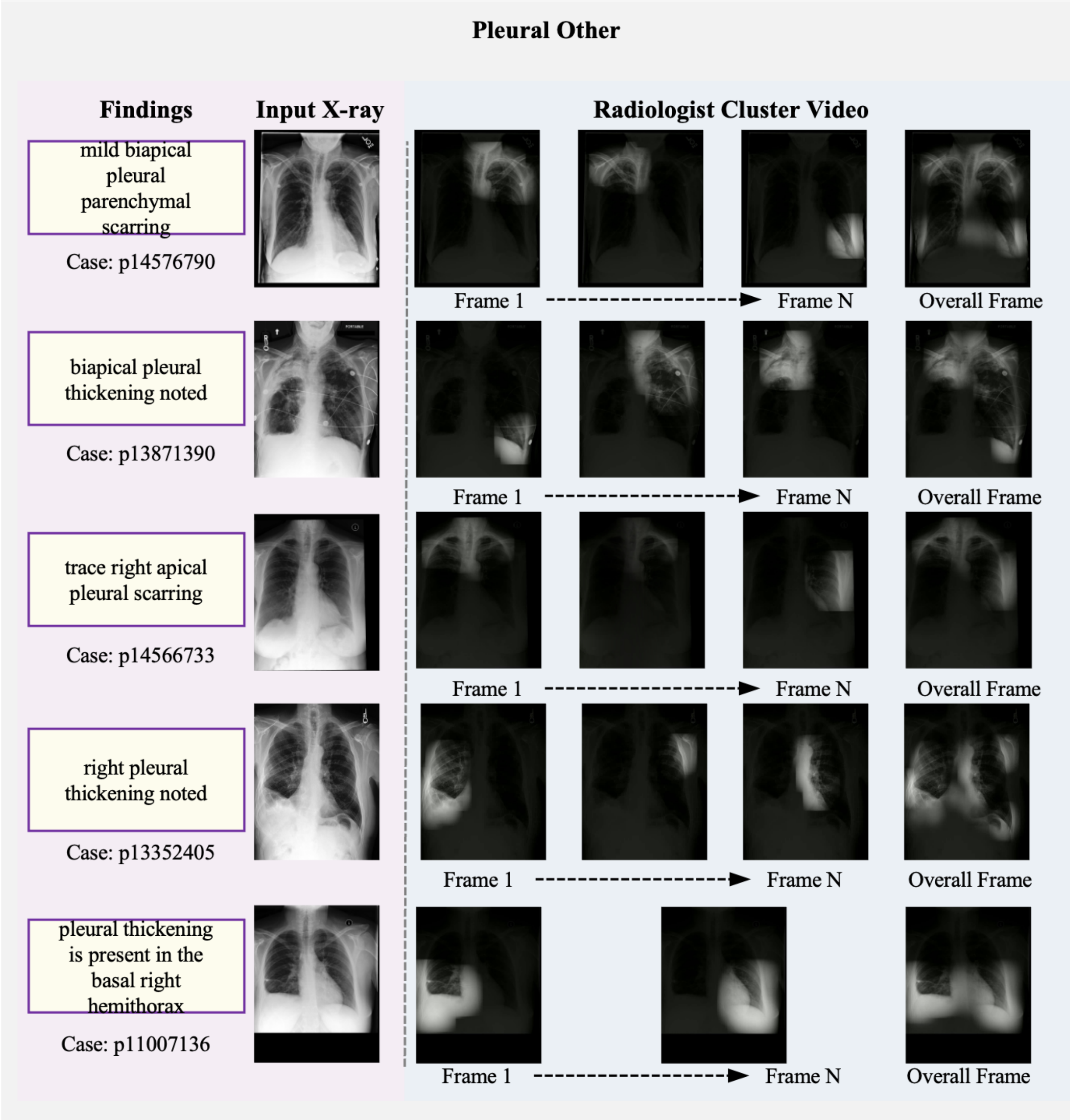

Supplementary Figure 16: **Gaze-guided attention videos of different training samples for other condition occurred in pleural space.**

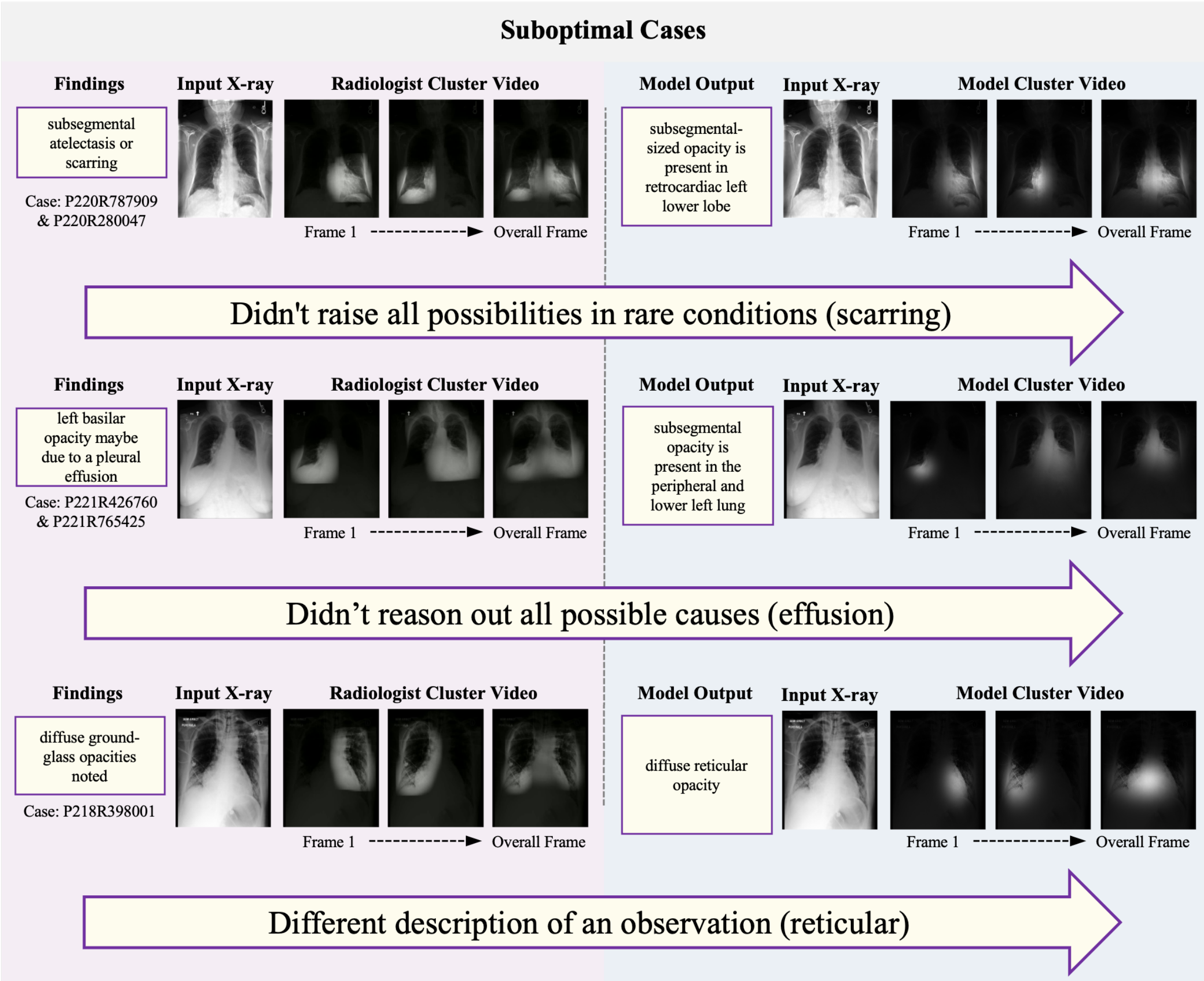

Supplementary Figure 17: **Suboptimal cases in GazeX examination compared to expert radiologists.** Three case studies were presented to investigate situations in which GazeX performs suboptimally. These cases demonstrated that, even when the model made generally correct observations, it failed to raise all possible diagnoses — particularly rare conditions with limited representation in the training data (e.g., scarring). Additionally, GazeX did not systematically reason through all potential causes of a given finding, and its descriptions of certain observations were slightly but meaningfully different from those provided by expert radiologists.

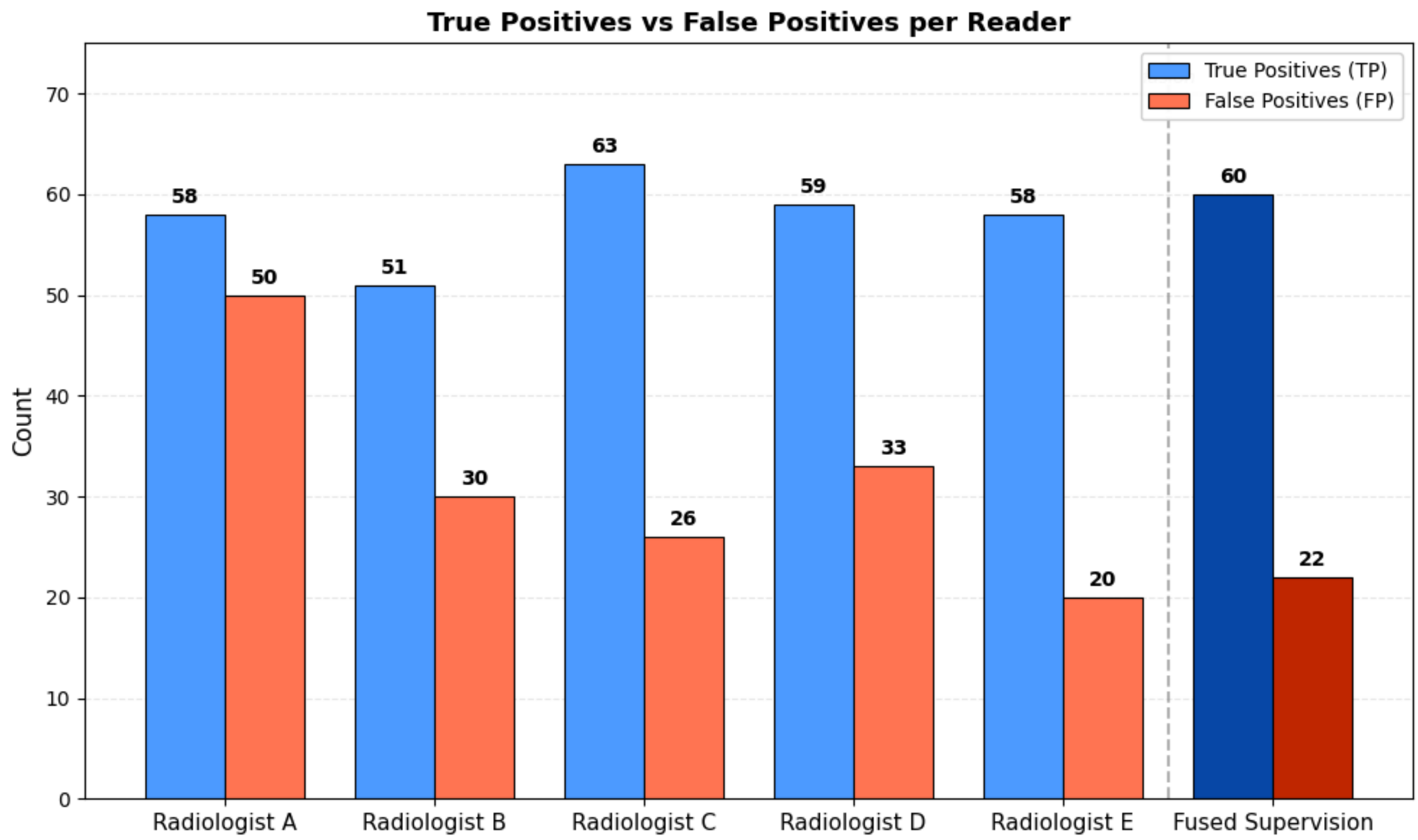

Supplementary Figure 18: **Ablation study on single-reader versus fused multi-reader gaze supervision.** Grouped bar chart comparing the number of true positives (TP, blue) and false positives (FP, orange) produced by six models trained under identical architectures and hyperparameters but with different gaze supervision signals: five models supervised by an individual radiologist and one model supervised by aggregating gaze maps across all five radiologists. Ground truth is defined by the majority vote of the five radiologists' diagnoses, following the protocol in Fig. 7c. The fused model attains a true-positive count (TP = 60) close to the most sensitive single reader (TP = 63), while simultaneously keeping false positives (FP = 22) close to the most specific reader (FP = 20). Performance of model trained with fused supervision outperform the average single-reader performance, demonstrating that fusion supervision improves robustness rather than averaging out informative search strategies.

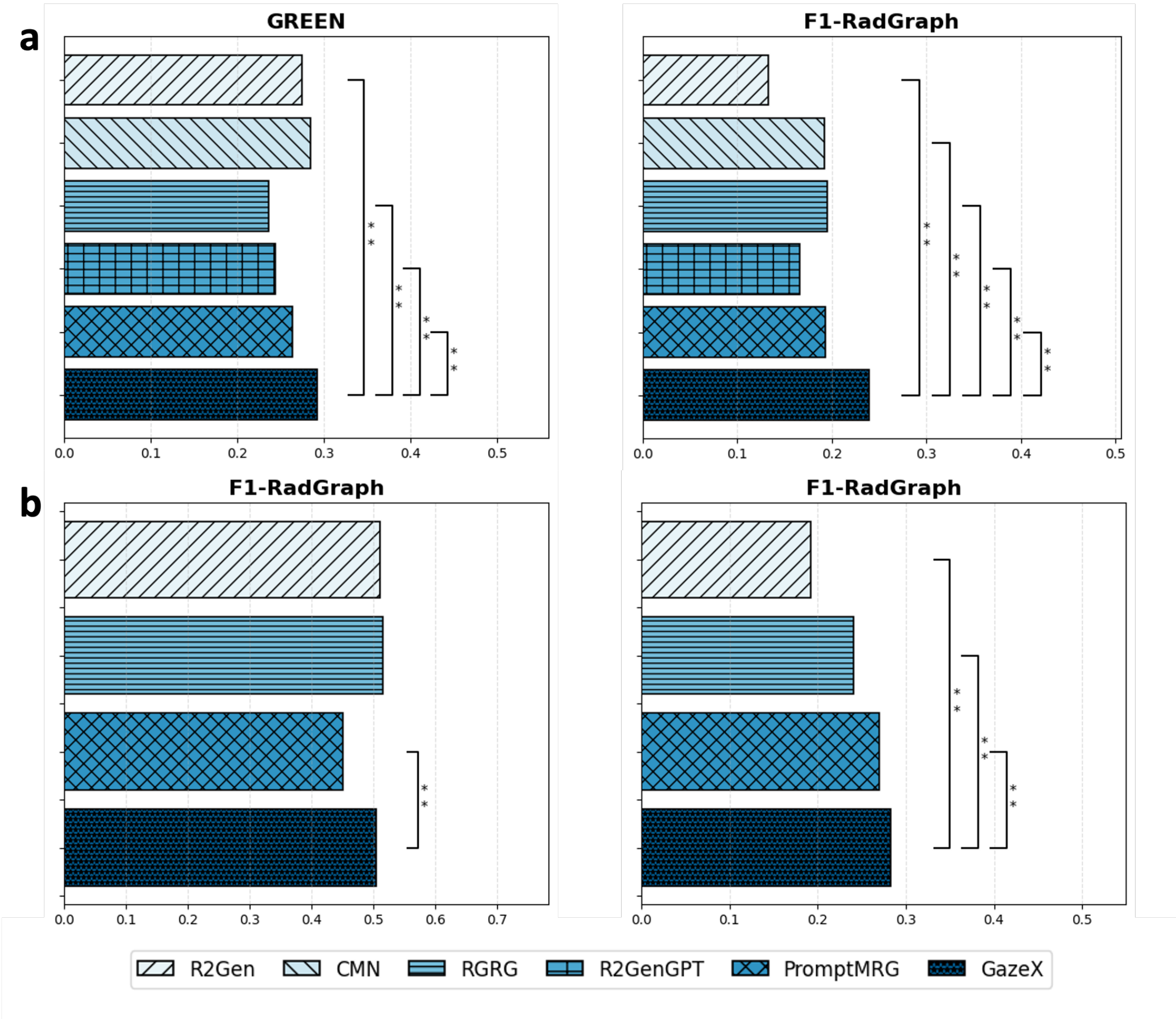

Supplementary Figure 19: **Comparison of different report generation methods across GREEN and RadGraph-F1.** Comparison of different report generation methods across GREEN [63] and RadGraph-F1 [64] in (a) MIMIC-CXR and (b) IU X-ray in zero shot setting. The Wilcoxon signed-rank test was performed to assess the statistical significance of the improvements compared to other models. ** indicates $p < 0.01$ and * indicates $p < 0.05$.

Supplementary Table 1: Comparison of different report generation methods across various evaluation metrics in MIMIC-CXR [1] and IU X-ray [2]. The best results are highlighted in **bold**. † indicates the result was reproduced.

Method	Year	BLEU-1	BLEU-2	BLEU-3	BLEU-4	METEOR	ROUGE-L	CheXbert
**Dataset: Mimicxr**								
R2Gen [46]†	2020	0.368	0.220	0.145	0.103	0.149	0.272	0.276
CMN [47]†	2021	0.353	0.213	0.140	0.098	0.162	0.279	0.278
MA [51]	2022	0.396	0.244	0.162	0.115	0.151	0.274	0.389
RGRG [48]†	2023	0.300	0.182	0.120	0.082	0.156	0.249	0.447
R2GenGPT [49]	2023	0.409	0.261	0.179	0.128	0.160	0.287	0.428
PromptMRG [50]	2024	0.398	–	–	0.112	0.157	0.268	**0.476**
STREAM [52]	2025	0.420	0.267	**0.184**	**0.133**	0.164	0.291	0.462
GazeX	2025	**0.427**	**0.277**	0.182	**0.133**	**0.187**	**0.315**	0.460
**Dataset: IU X-ray**								
R2Gen [46]	2020	0.475	0.304	0.219	0.170	0.187	0.371	–
CMN [47]	2021	0.475	0.309	0.222	0.170	0.191	0.375	–
MA [51]	2022	0.500	0.328	0.230	0.170	0.213	0.386	–
R2GenGPT [49]	2023	0.491	0.323	0.234	0.180	0.211	0.376	–
PromptMRG [50]	2024	0.401	–	–	0.098	0.160	0.281	–
STREAM [52]	2025	0.499	**0.333**	**0.238**	**0.178**	0.213	0.377	–
GazeX	2025	**0.502**	**0.333**	0.234	0.170	**0.227**	**0.398**	–
**Dataset: IU X-ray Zero Shot**								
R2Gen [46]	2020	0.325	–	–	0.059	0.131	0.253	0.136
RGRG [48]†	2023	0.266	–	–	0.063	0.146	0.180	0.180
PromptMRG [50]	2024	0.401	–	–	0.098	0.160	0.281	0.211
GazeX	2025	**0.423**	0.248	0.157	**0.103**	**0.181**	**0.314**	**0.220**

Supplementary Table 2: Comparison of different medical foundation models in X-ray VQA task [3]. The best results are highlighted in **bold**. † indicates the result was reproduced.

Method	Year	Abnormality	Presence	Location	Level	Type	View	Combined
**Dataset: Medical-CXR-VQA**								
LLava−Med [53]†	2023	0.192	0.630	0.052	0.025	0.055	0.700	0.314
RadFM [23]†	2023	0.367	0.629	0.052	0.079	0.049	0.521	0.343
HuatuoGPT−Vision [24]†	2024	0.175	0.244	0.001	0.013	0.043	0.319	0.151
HuatuoGPT−Vision−32B [24]†	2024	0.182	0.248	0.000	0.022	0.053	0.584	0.202
Bio−Medical−MultiModal−Llama [54] †	2024	0.182	0.596	0.057	0.078	0.068	0.625	0.303
Medical-CXR-VQA [3]†	2024	0.459	0.891	0.403	0.521	0.267	0.743	0.580
Medgemma−4b−it [55]†	2025	0.570	0.579	0.250	0.520	**0.618**	0.730	0.546
Lingshu−7B [56]†	2025	0.454	0.713	0.067	0.079	0.042	0.961	0.440
Lingshu−32B [56]†	2025	0.436	0.703	0.048	0.259	0.054	0.955	0.457
GazeX−2B	2025	**0.594**	**0.902**	**0.435**	**0.530**	0.273	**0.994**	**0.651**

Supplementary Table 3: Comparison of different visual grounding methods across various evaluation metrics in MS-CXR dataset [4].

Method	Year	mIOU	mAP
ChEX [57]	2025	0.475	0.444
TransVG [58]	2025	0.535	0.440
BioVIL [4]	2025	0.285	0.186
OmniFM−DR [59]	2025	0.462	–
GazeX	2025	0.517	0.462

Supplementary Table 4: Legends for the video files provided.

Video Name	Legend
P127R581185.mp4	Gaze-guided attention video training sample for atelectasis
P300R038481.mp4	Gaze-guided attention video training sample for atelectasis
P300R264915.mp4	Gaze-guided attention video training sample for atelectasis
P300R429412.mp4	Gaze-guided attention video training sample for atelectasis
P300R482328.mp4	Gaze-guided attention video training sample for atelectasis
P108R988542.mp4	Gaze-guided attention video training sample for cardiomegaly
P300R084617.mp4	Gaze-guided attention video training sample for cardiomegaly
P300R181623.mp4	Gaze-guided attention video training sample for cardiomegaly
P300R510107.mp4	Gaze-guided attention video training sample for cardiomegaly
P300R904453.mp4	Gaze-guided attention video training sample for cardiomegaly
P300R117470.mp4	Gaze-guided attention video training sample for consolidation
P300R166837.mp4	Gaze-guided attention video training sample for consolidation
P300R511335.mp4	Gaze-guided attention video training sample for consolidation
P300R746426.mp4	Gaze-guided attention video training sample for consolidation
P300R859980.mp4	Gaze-guided attention video training sample for consolidation
P300R268663.mp4	Gaze-guided attention video training sample for edema
P300R274134.mp4	Gaze-guided attention video training sample for edema
P300R521261.mp4	Gaze-guided attention video training sample for edema
P300R728475.mp4	Gaze-guided attention video training sample for edema
P300R834802.mp4	Gaze-guided attention video training sample for edema
P300R137578.mp4	Gaze-guided attention video training sample for enlarged cardiomediastinum
P300R423838.mp4	Gaze-guided attention video training sample for enlarged cardiomediastinum
P300R440180.mp4	Gaze-guided attention video training sample for enlarged cardiomediastinum
P300R699827.mp4	Gaze-guided attention video training sample for enlarged cardiomediastinum
P300R848686.mp4	Gaze-guided attention video training sample for enlarged cardiomediastinum
P300R040793.mp4	Gaze-guided attention video training sample for fracture
P300R170238.mp4	Gaze-guided attention video training sample for fracture
P300R328250.mp4	Gaze-guided attention video training sample for fracture
P300R735400.mp4	Gaze-guided attention video training sample for fracture
P300R944758.mp4	Gaze-guided attention video training sample for fracture
P116R051205.mp4	Gaze-guided attention video training sample for lung lesion
P147R695031.mp4	Gaze-guided attention video training sample for lung lesion
P300R458083.mp4	Gaze-guided attention video training sample for lung lesion
P300R912852.mp4	Gaze-guided attention video training sample for lung lesion
P300R928658.mp4	Gaze-guided attention video training sample for lung lesion
P153R647043.mp4	Gaze-guided attention video training sample for lung opacity
P300R296391.mp4	Gaze-guided attention video training sample for lung opacity
P300R746512.mp4	Gaze-guided attention video training sample for lung opacity
P300R892488.mp4	Gaze-guided attention video training sample for lung opacity
P300R946762.mp4	Gaze-guided attention video training sample for lung opacity
P140R411353.mp4	Gaze-guided attention video training sample for pleural effusion
P155R307812.mp4	Gaze-guided attention video training sample for pleural effusion
P300R263650.mp4	Gaze-guided attention video training sample for pleural effusion
P300R663705.mp4	Gaze-guided attention video training sample for pleural effusion
P300R871821.mp4	Gaze-guided attention video training sample for pleural effusion
P300R133668.mp4	Gaze-guided attention video training sample for pleural other
P300R317775.mp4	Gaze-guided attention video training sample for pleural other
P300R324597.mp4	Gaze-guided attention video training sample for pleural other
P300R406052.mp4	Gaze-guided attention video training sample for pleural other
P300R427400.mp4	Gaze-guided attention video training sample for pleural other
P300R121261.mp4	Gaze-guided attention video training sample for pneumothorax
P300R336111.mp4	Gaze-guided attention video training sample for pneumothorax

Continued on next page

Video Name	Legend
P300R899329.mp4	Gaze-guided attention video training sample for pneumothorax
P300R920997.mp4	Gaze-guided attention video training sample for pneumothorax
P300R988859.mp4	Gaze-guided attention video training sample for pneumothorax
P300R112768.mp4	Gaze-guided attention video training sample for support apparatus
P300R208749.mp4	Gaze-guided attention video training sample for support apparatus
P300R553198.mp4	Gaze-guided attention video training sample for support apparatus
P300R697795.mp4	Gaze-guided attention video training sample for support apparatus
P300R794529.mp4	Gaze-guided attention video training sample for support apparatus

References

[1] Johnson, A. E. *et al.* Mimic-cxr, a de-identified publicly available database of chest radiographs with free-text reports. *Scientific data* **6**, 317 (2019).

[2] Demner-Fushman, D. *et al.* Preparing a collection of radiology examinations for distribution and retrieval. *Journal of the American Medical Informatics Association* **23**, 304–310 (2016).

[3] Hu, X. *et al.* Interpretable medical image visual question answering via multi-modal relationship graph learning. *Medical Image Analysis* **97**, 103279 (2024).

[4] Boecking, B. *et al.* Making the most of text semantics to improve biomedical vision–language processing. In *European conference on computer vision*, 1–21 (Springer, 2022).

[5] Zhang, Y., Jiang, H., Miura, Y., Manning, C. D. & Langlotz, C. P. Contrastive learning of medical visual representations from paired images and text. In *Machine learning for healthcare conference*, 2–25 (PMLR, 2022).

[6] Lee, S., Youn, J., Kim, H., Kim, M. & Yoon, S. H. Cxr-llava: a multimodal large language model for interpreting chest x-ray images. *European Radiology* 1–13 (2025).

[7] Yuan, J. Contrastive multimodal learning for chest x-ray analysis: Leveraging text and image synergy. In *2025 8th International Conference on Advanced Algorithms and Control Engineering (ICAACE)*, 2523–2527 (IEEE, 2025).

[8] Chen, T., Kornblith, S., Norouzi, M. & Hinton, G. A simple framework for contrastive learning of visual representations. In *International conference on machine learning*, 1597–1607 (PmLR, 2020).

[9] Jing, D. *et al.* Fineclip: Self-distilled region-based clip for better fine-grained understanding. In *Advances in Neural Information Processing Systems*, vol. 37, 27896–27918 (Curran Associates, Inc., 2024).

[10] Radford, A. *et al.* Learning transferable visual models from natural language supervision. In *International conference on machine learning*, 8748–8763 (PmLR, 2021).

[11] Touvron, H. *et al.* Llama: Open and efficient foundation language models. *arXiv preprint arXiv:2302.13971* (2023).

[12] Tanno, R. *et al.* Collaboration between clinicians and vision–language models in radiology report generation. *Nature Medicine* **31**, 599–608 (2025).

[13] Gu, D., Gao, Y., Zhou, Y., Zhou, M. & Metaxas, D. Radalign: Advancing radiology report generation with vision-language concept alignment. In *Medical Image Computing and Computer Assisted Intervention – MICCAI 2025*, 484–494 (2025).

[14] Park, J., Kim, S., Yoon, B., Hyun, J. & Choi, K. M4cxr: Exploring multitask potentials of multimodal large language models for chest x-ray interpretation. *IEEE Transactions on Neural Networks and Learning Systems* (2025).

[15] Chen, Z. *et al.* Medical phrase grounding with region-phrase context contrastive alignment. In *International Conference on Medical Image Computing and Computer-Assisted Intervention*, 371–381 (Springer, 2023).

[16] Wu, C., Zhang, X., Zhang, Y., Wang, Y. & Xie, W. Medklip: Medical knowledge enhanced language-image pre-training for x-ray diagnosis. In *Proceedings of the IEEE/CVF international conference on computer vision*, 21372–21383 (2023).

[17] Zhang, K. *et al.* A generalist vision–language foundation model for diverse biomedical tasks. *Nature Medicine* 1–13 (2024).

[18] Çallı, E., Sogancioglu, E., Van Ginneken, B., van Leeuwen, K. G. & Murphy, K. Deep learning for chest x-ray analysis: A survey. *Medical image analysis* **72**, 102125 (2021).

[19] Zhou, Y. *et al.* A foundation model for generalizable disease detection from retinal images. *Nature* **622**, 156–163 (2023).

[20] Huang, Z., Bianchi, F., Yuksekgonul, M., Montine, T. J. & Zou, J. A visual–language foundation model for pathology image analysis using medical twitter. *Nature medicine* **29**, 2307–2316 (2023).

[21] Christensen, M., Vukadinovic, M., Yuan, N. & Ouyang, D. Vision–language foundation model for echocardiogram interpretation. *Nature Medicine* **30**, 1481–1488 (2024).

[22] Ma, D., Pang, J., Gotway, M. B. & Liang, J. A fully open ai foundation model applied to chest radiography. *Nature* 1–11 (2025).

[23] Wu, C. *et al.* Towards generalist foundation model for radiology by leveraging web-scale 2d&3d medical data. *Nature Communications* **16**, 7866 (2025).

[24] Chen, J. *et al.* Huatuogpt-vision, towards injecting medical visual knowledge into multimodal llms at scale. *arXiv preprint arXiv:2406.19280* (2024).

[25] Pan, J. *et al.* Medvlm-r1: Incentivizing medical reasoning capability of vision-language models (vlms) via reinforcement learning. *arXiv preprint arXiv:2502.19634* (2025).

[26] Saab, K. *et al.* Capabilities of gemini models in medicine. *arXiv preprint arXiv:2404.18416* (2024).

[27] Chen, Z. *et al.* Chexagent: Towards a foundation model for chest x-ray interpretation. *arXiv preprint arXiv:2401.12208* (2024).

[28] Yan, S. *et al.* Vigor: Improving visual grounding of large vision language models with fine-grained reward modeling. In *Computer Vision – ECCV 2024*, 37–53 (Springer Nature Switzerland, Cham, 2025).

[29] Jing, P. *et al.* Reason like a radiologist: Chain-of-thought and reinforcement learning for verifiable report generation. *arXiv preprint arXiv:2504.18453* (2025).

[30] Nobel, J. M., van Geel, K. & Robben, S. G. Structured reporting in radiology: a systematic review to explore its potential. *European radiology* **32**, 2837–2854 (2022).

[31] Hosny, A., Parmar, C., Quackenbush, J., Schwartz, L. H. & Aerts, H. J. Artificial intelligence in radiology. *Nature Reviews Cancer* **18**, 500–510 (2018).

[32] Holmqvist, K. *et al.* *Eye tracking: A comprehensive guide to methods and measures* (oup Oxford, 2011).

[33] Van der Gijp, A. *et al.* How visual search relates to visual diagnostic performance: a narrative systematic review of eye-tracking research in radiology. *Advances in Health Sciences Education* **22**, 765–787 (2017).

[34] Lévêque, L., Bosmans, H., Cockmartin, L. & Liu, H. State of the art: Eye-tracking studies in medical imaging. *Ieee Access* **6**, 37023–37034 (2018).

[35] Kundel, H. L., Nodine, C. F., Conant, E. F. & Weinstein, S. P. Holistic component of image perception in mammogram interpretation: gaze-tracking study. *Radiology* **242**, 396–402 (2007).

[36] Swensson, R. G. A two-stage detection model applied to skilled visual search by radiologists. *Perception & Psychophysics* **27**, 11–16 (1980).

[37] Christopher Clarke, A. D. *Chest X-rays for Medical Students* (Wiley–Blackwell, 2011).

[38] Kool, D. R. & Blickman, J. G. Advanced trauma life support®. abcde from a radiological point of view. *Emergency radiology* **14**, 135–141 (2007).

[39] Wang, S. *et al.* Learning better contrastive view from radiologist's gaze. *Pattern Recognition* **162**, 111350 (2025).

[40] Ma, C. *et al.* Eye-gaze guided multi-modal alignment framework for radiology. *CoRR* (2024).

[41] Xie, J. *et al.* Integrating eye tracking with grouped fusion networks for semantic segmentation on mammogram images. *IEEE Transactions on Medical Imaging* (2024).

[42] Ester, M., Kriegel, H.-P., Sander, J. & Xu, X. A density-based algorithm for discovering clusters in large spatial databases with noise. In *Proceedings of the Second International Conference on Knowledge Discovery and Data Mining*, KDD'96, 226–231 (AAAI Press, 1996).

[43] Smit, A. *et al.* Combining automatic labelers and expert annotations for accurate radiology report labeling using BERT. In *Proceedings of the 2020 Conference on Empirical Methods in Natural Language Processing (EMNLP)*, 1500–1519 (Association for Computational Linguistics, 2020).

[44] Wang, P. *et al.* Qwen2-vl: Enhancing vision-language model's perception of the world at any resolution. *arXiv preprint arXiv:2409.12191* (2024).

[45] Hu, E. J. *et al.* LoRA: Low-rank adaptation of large language models. In *International Conference on Learning Representations* (2022).

[46] Chen, Z., Song, Y., Chang, T.-H. & Wan, X. Generating radiology reports via memory-driven transformer. In *Proceedings of the 2020 Conference on Empirical Methods in Natural Language Processing* (2020).

[47] Chen, Z., Shen, Y., Song, Y. & Wan, X. Cross-modal memory networks for radiology report generation. In *Proceedings of the 59th Annual Meeting of the Association for Computational Linguistics*, 5904–5914 (Association for Computational Linguistics, 2021).

[48] Tanida, T., Müller, P., Kaissis, G. & Rueckert, D. Interactive and explainable region-guided radiology report generation. In *Proceedings of the IEEE/CVF Conference on Computer Vision and Pattern Recognition*, 7433–7442 (2023).

[49] Wang, Z., Liu, L., Wang, L. & Zhou, L. R2gengpt: Radiology report generation with frozen llms. *Meta-Radiology* **1**, 100033 (2023).

[50] Jin, H., Che, H., Lin, Y. & Chen, H. Promptmrg: diagnosis-driven prompts for medical report generation. In *Proceedings of the Thirty-Eighth AAAI Conference on Artificial Intelligence* (AAAI Press, 2024).

[51] Wang, Z., Han, H., Wang, L., Li, X. & Zhou, L. Automated radiographic report generation purely on transformer: A multicriteria supervised approach. *IEEE Transactions on Medical Imaging* **41**, 2803–2813 (2022).

[52] Yang, Y. *et al.* Spatio-temporal and retrieval-augmented modeling for chest x-ray report generation. *IEEE Transactions on Medical Imaging* **44**, 2892–2905 (2025).

[53] Li, C. *et al.* Llava-med: training a large language-and-vision assistant for biomedicine in one day. In *Proceedings of the 37th International Conference on Neural Information Processing Systems*, NIPS '23 (Curran Associates Inc., 2023).

[54] ContactDoctor. Contactdoctor-bio-medical-multimodal-llama-3-8b-v1: A high-performance biomedical multimodal llm (2024).

[55] Google. Medgemma hugging face (2025). Accessed: [Insert Date Accessed, e.g., 2025-05-20].

[56] Xu, W. *et al.* Lingshu: A generalist foundation model for unified multimodal medical understanding and reasoning. *arXiv preprint arXiv:2506.07044* (2025).

[57] Müller, P., Kaissis, G. & Rueckert, D. Chex: Interactive localization and region description in chest x-rays. In *European Conference on Computer Vision*, 92–111 (Springer, 2024).

[58] Deng, J., Yang, Z., Chen, T., Zhou, W. & Li, H. Transvg: End-to-end visual grounding with transformers. In *Proceedings of the IEEE/CVF International Conference on Computer Vision*, 1769–1779 (2021).

[59] Xu, L. *et al.* Learning a multi-task transformer via unified and customized instruction tuning for chest radiograph interpretation. *arXiv preprint arXiv:2311.01092* (2023).

[60] Li, Y. *et al.* Thinking with gaze: Sequential eye-tracking as visual reasoning supervision for medical vlms. *arXiv preprint arXiv:2603.06697* (2026).

[61] Beckmann, D. & Risse, B. Fixationformer: Direct utilization of expert gaze trajectories for chest x-ray classification. *arXiv preprint arXiv:2603.22939* (2026).

[62] Lvov, D. & Pershin, I. From human attention to diagnosis: Semantic patch-level integration of vision-language models in medical imaging. In *Advances in Neural Information Processing Systems (NeurIPS)* (2025).

[63] Ostmeier, S. *et al.* GREEN: Generative radiology report evaluation and error notation. *arXiv preprint arXiv:2405.03595* (2024). 2405.03595.

[64] Jain, S. *et al.* RadGraph: Extracting clinical entities and relations from radiology reports. *arXiv preprint arXiv:2106.14463* (2021). 2106.14463.
